# Population Genetics of *Lactobacillus sakei* Reveals Three Lineages with Distinct Evolutionary Histories

**DOI:** 10.1371/journal.pone.0073253

**Published:** 2013-09-19

**Authors:** Stéphane Chaillou, Isabelle Lucquin, Afef Najjari, Monique Zagorec, Marie-Christine Champomier-Vergès

**Affiliations:** 1 INRA, UMR1319 Micalis, Jouy-en-Josas, France; 2 AgroParisTech, UMR1319 Micalis, Jouy-en-Josas, France; 3 Laboratoire de Microbiologie et Biomolécules Actives, Faculté des Sciences de Tunis, Tunis, Tunisia; Catalan Institute for Water Research (ICRA), Spain

## Abstract

*Lactobacillus sakei* plays a major role in meat fermentation and in the preservation of fresh meat. The large diversity of *L. sakei* strains represents a valuable and exploitable asset in the development of a variety of industrial applications; however, an efficient method to identify and classify these strains has yet to be developed. In this study, we used multilocus sequence typing (MLST) to analyze the polymorphism and allelic distribution of eight loci within an *L. sakei* population of 232 strains collected worldwide. Within this population, we identified 116 unique sequence types with an average pairwise nucleotide diversity per site (*π*) of 0.13%. Results from Structure, goeBurst, and ClonalFrame software analyses demonstrated that the *L. sakei* population analyzed here is derived from three ancestral lineages, each of which shows evidence of a unique evolutionary history influenced by independent selection scenarios. However, the signature of selective events in the contemporary population of isolates was somewhat masked by the pervasive phenomenon of homologous recombination. Our results demonstrate that lineage 1 is a completely panmictic subpopulation in which alleles have been continually redistributed through the process of intra-lineage recombination. In contrast, lineage 2 was characterized by a high degree of clonality. Lineage 3, the earliest-diverging branch in the genealogy, showed evidence of both clonality and recombination. These evolutionary histories strongly indicate that the three lineages may correspond to distinct ecotypes, likely linked or specialized to different environmental reservoirs. The MLST scheme developed in this study represents an easy and straightforward tool that can be used to further analyze the population dynamics of *L. sakei* strains in food products.

## Introduction

*Lactobacillus sakei* is a psychrotrophic lactic acid bacterium highly prevalent in various types of meat products. This microorganism flourishes at low temperatures and frequently becomes one of the dominant flora during cold storage. Because of this, *L. sakei* plays a major role in meat fermentation during the production of traditional or industrial dry sausages [1]–[3] and also possesses properties useful for the preservation of fresh meat [4]–[6]. The positive influence of *L. sakei* on food preservation can be explained mainly by its physiological and genomic adaptations to growth on meat products, which result in it outcompeting other disease- or spoilage-causing microorganisms [7]. At present, both academic and industrial researchers are attempting to develop methods to screen new strains and to exploit *L. sakei*'s strain diversity in the development of innovative biotechnological processes. As a result, there is a great need for accurate tools that can identify and assess the diversity of strains, improve laboratory collection management, and aid in efficient bacterial traceability in meat products.

Several studies attempting to characterize the genetic diversity of *L. sakei* have been already published. The most important classification produced so far resulted in the separation of *L. sakei* strains into two subspecies: *L. sakei* ssp. *sakei* and *L. sakei* ssp. *carnosus* [8]. This subspecies classification was based on the soluble cell protein profiles of 50 strains from a single laboratory collection [9]; it was then further validated by the results of a randomly amplified polymorphic DNA (RAPD) analysis carried out on a subset of 24 strains. Subsequently, an alternative, broader RAPD analysis was carried out on a hundred strains from various collections; it revealed a larger number of distinct profiles but no clear subspecies separation [10]. The contrasting results of these two studies suggest that a binary clustering of strains into two taxonomic subspecies might not accurately or sufficiently represent the evolution of diversity within this species.

More recently, the classification of *L. sakei* into two subspecies was further challenged by genomic comparisons of a set of 73 strains selected from 14 laboratory collections [11]. PCR binary typing of 60 variable genes revealed the presence of 10 genotypic clusters, and pulse-field gel electrophoresis revealed that chromosome size varied by up to 25% between strains. Furthermore, using 2D protein gel electrophoresis, we were able to demonstrate that the division of *L. sakei* into two subspecies was only supported by the migrating variation of four isoforms of GapA (glyceraldehyde 3P dehydrogenase), one of the most abundant soluble proteins when cells are grown in laboratory conditions. Given these considerations, we have come to the conclusion that classification methods based on soluble cell protein profiles may lack sufficient power to resolve the large intraspecific diversity of *L. sakei*, and thus decided that a phylogenetic analysis using multilocus sequence typing (MLST) was required to clearly and unambiguously describe *L. sakei* population structure.

MLST consists of analyzing the nucleotide and allelic polymorphism of a set of 6 to 10 housekeeping genes; it is the “gold standard” technique for studying microbial population biology [12]. MLST has been widely used for epidemiological and evolutionary analyses of bacterial pathogens and has also been applied to studies of food bacteria. Several MLST schemes have been published in recent years for lactic acid bacteria, including *Lactobacillus plantarum* [13], *Lactobacillus delbrueckii* [14], *Lactobacillus casei* [15], [16], *Lactobacillus sanfranciscensis* [17], *Lactobacillus salivarius* [18], *Lactobacillus reuteri* [19], *Oenococcus oeni* [19], *Lactococcus lactis* [20], and *Streptococcus thermophilus* [21]. These studies involved only a moderate number of isolates (from 20 to 100) but revealed that MLST was an efficient strain-typing method with the potential to provide information about the evolutionary histories of food-borne bacteria.

Here, we report the results of an MLST analysis investigating the population structure of *L. sakei*. We used a set of 232 strains, encompassing many isolation sources and laboratory collections throughout the world. We show that the species has a complex population structure, which is divided into three lineages with distinct evolutionary histories. This study provides a much clearer view of strain diversity that will be valuable in re-evaluating the species' taxonomic description. We also propose various hypotheses regarding the possible ecological forces driving the evolution of this species.

## Results

### Descriptive analysis of nucleotide sequence data

We developed our MLST scheme using a top-down strategy (see Materials and Methods) that resulted in the selection of eight genes from the *L. sakei* reference genome 23K. These genes included three housekeeping genes (*recA*, *tuf*, *rpoB*), three catabolic genes (*pepV*, *glpF*, *ldhL*), one putative anabolic gene (*hemN*), and one stress-response gene (*dnaK*). Partial sequencing of the eight loci revealed 158 single-nucleotide polymorphisms (SNPs) across the 3,334 bp of the concatenated sequences (Table 1). The population of 232 strains (described in Table S1) contained a total of 116 unique sequence types (STs). The average pairwise nucleotide diversity per site (*π*) was 0.13% for all the concatenated sequences taken together and ranged from 0.04% (*ldhL*, *pepV*) to 0.26% (*recA*) for individual loci. The number of alleles found at each locus ranged from 8 (*pepV*) to 21 (*dnaK*, *hemN*), a 2.6-fold difference in allelic diversity. Likewise, when we looked at the number of polymorphic sites (*S*), we found between 9 (*ldhL*) and 37 (*hemN*) at each locus, a 4.1-fold maximum difference between pairs of loci. Altogether, these data demonstrate that none of the eight loci accounted for more than 25% of the overall nucleotide or allelic diversity, indicating that the developed MLST scheme was well balanced and complied with proper MLST scheme development practices [12].

**Table 1:** Nucleotide sequence analysis of the eight MLST loci.

Gene	Locus position (kb)	Lenght (bp) of the sequenced fragment	GC%	*S*	π/site	Tajima *D*	Nb of alleles
*pepV*	431	540	47.0	12 (0)	0.0044	0.136	8
*recA*	504	399	38.8	30 (3)	0.0265	2.452*	13
*glpF*	655	399	44.2	13 (1)	0.0091	1.286	12
*Tuf*	1057	330	39.0	14 (8)	0.0134	1.548	9
*dnaK*	1214	519	45.1	24 (2)	0.0147	1.513	21
*hemN*	1220	369	42.2	37 (3)	0.0256	0.894	21
*ldhL*	1593	441	41.2	9 (3)	0.0046	0.481	9
*rpoB*	1747	369	39.5	19 (3)	0.0106	0.122	12
Concatenated		3334	41.2	158 (23)	0.0131	1.361	116

Locus position: coordinates in kb based on *L. sakei* 23K chromosome.

*S:* number of polymorphic sites (non-synonymous sites are shown in brackets).

π/site : Average pairwise nucleotide difference per site.

*D:* Tajima's *D* statistical value based on the population of 232 strains (significant deviation from 0 and from standard neutral model is marked by an *, p<0.05).

The number of non-synonymous substitutions was low (*n = *23) in comparison to the total number of SNPs (*n = *158); this result indicated that most of the polymorphisms were selectively neutral and that the target loci were likely subject to stabilizing selection. Only the housekeeping gene *tuf* deviated significantly from this general trend: almost 60% of its mutations were non-synonymous, an outcome indicative of the presence of strong diversifying selection at this locus. Tajima's *D* test [23] was used to evaluate deviation from the standard neutral model of evolution; positive *D* values were found for all loci, although they were only statistically significant for *recA*. Nevertheless, given that the concatenated sequence had a *D* value of 1.36, this trend was relatively pronounced and is likely a signature of balancing selection in a bacterial species that is clearly structured into genetically distinct subpopulations.

### Population is structured into 3 subpopulations

**Figure 1:**
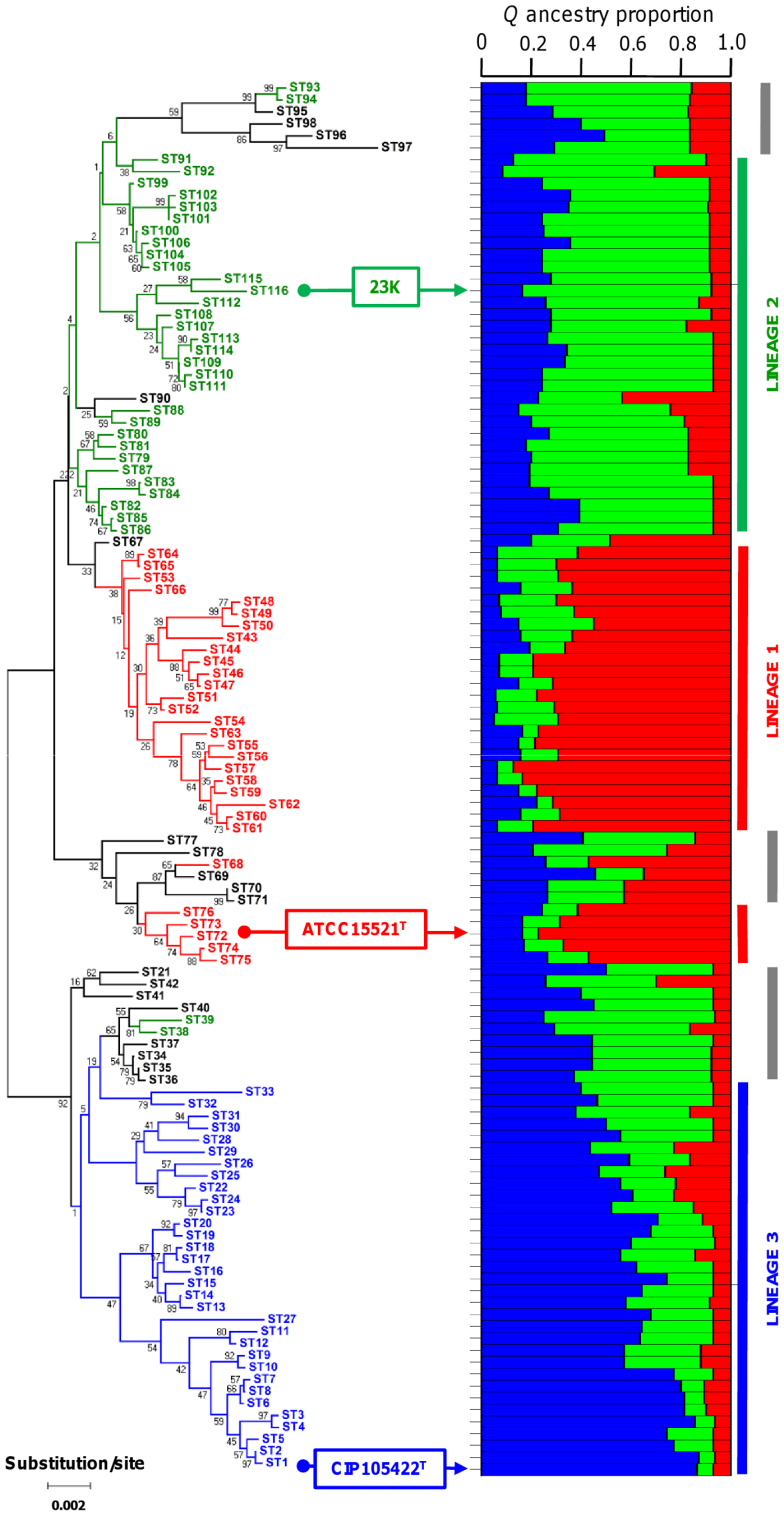
Ancestry and phylogenetic tree of 116 *L. sakei* STs. (right) Proportion of genetic material derived from each of three subpopulations for each ST as inferred by Structure (assuming *K* = 3 populations and applying the linkage model). Ancestral subpopulations are colored in red (lineage 1), green (lineage 2), and blue (lineage 3), respectively. Each bar (stacked vertically) represents one of 116 STs, ordered on the y axis by their positions in the NJ tree. The vertical thick gray lines show clusters of STs with mixed population origins. (left) Phylogenetic NJ tree obtained from the concatenated sequences of the 116 STs. Bootstrap values are indicated for all branches. STs are colored according to their affiliation to one of the three lineages; admixed STs are in black. The positions of the two *L. sakei* subspecies type strains and of strain 23K are indicated by arrows.

We attempted to statistically estimate the number of ancestral subpopulations (*K*) within the genetic population of *L. sakei* strains using Structure [24] with the linkage model [25]. Several values of *K* were tested, from *K = *2 (separation into two subspecies as indicated by the results of Torriani et al. [8]) to *K = *10 (possible separation into ten genotypic clusters as suggested by our previous study [11]). We found maximal posterior probability for *K = *3: the genetic diversity of *L. sakei* was divided into 3 ancestral populations and most individual STs had a high likelihood of affiliation with this model of ancestry (see Materials & Methods section). Hereafter, we use the term “lineage” to describe the subpopulations (identified as 1, 2, or 3) inferred by this Bayesian approach. For each ST, the average proportion of genetic material derived from each lineage (*Q*) is shown in Figure 1. We found that the type strain of each of the two previously defined *L. sakei* subspecies displayed affinity to a different lineage: over 80% of the genetic material of *L. sakei* ssp. *sakei* (strain ATCC15521^T^) was derived from lineage 1, while the same was true of ssp. *carnosus* (strain CIP105422^T^) and lineage 3. However, the reference strain 23K, the only strain for which genome sequencing has been carried out [7], appeared to be mainly descended from lineage 2.

As shown in Figure 1, the branching pattern of a neighbor-joining (NJ) tree constructed using the concatenated sequences was consistent with the hypothesis of three ancestral lineages. However, the topology generated by the NJ method was not well supported; only 20% of branches had a bootstrap value greater than 70, and the average value was quite low (55). A similar tree was obtained using maximum-likelihood method (data not shown). The consistently low bootstrap values were representative of the inherently incongruent phylogenetic signals in the NJ topology, especially in the branches connecting lineage 1 and 2. A compatibility matrix produced by the Reticulate program [26] also indicated that the 8 loci delivered a pairwise incongruent signal (Figure S1). Further Structure analysis revealed that some STs contained a high degree of admixture (see the gray zones highlighted in Figure 1), a factor that possibly contributed to the overall poor support values of the NJ topology. For example, if we set the threshold for the assignment of an ST to a lineage at *Q*>0.5, about 90% of the population was evenly distributed into the three lineages. However, under these conditions, 19 STs remained unaffiliated with any lineage because they contained a higher degree of admixture (Table 2). When we used Structure to analyze the net nucleotide distance between lineages, we obtained results consistent with the NJ topology: the distance between lineage 1 and lineage 2 was shorter than that between 1 and 3 or 2 and 3. The largest net nucleotide distance was found between lineages 2 and 3.

**Table 2:** Comparative analysis of the three lineages using Structure linkage model.

	Affiliation of 116 STs based on Q ancestry proportion >0.5			Net nucleotide distance^1^		
	Nb of STs	Nb of strains	% of population	Lineage1	Lineage2	Lineage3
Lineage1	30	38	16.4	-	0.241	0.320
Lineage2	35	104	44.8	0.241	-	0.413
Lineage3	32	68	29.3	0.320	0.413	-
Admixed	19	22	9.5	NA	NA	NA

NA : not applicable.

1 Net nucleotide distance as inferred by STRUCTURE represents the allele frequency divergence among clusters. It is the average probability that a pair of alleles, one each from the 2 populations compared are different.

### Degree of clonality differs among lineages

**Figure 2:**
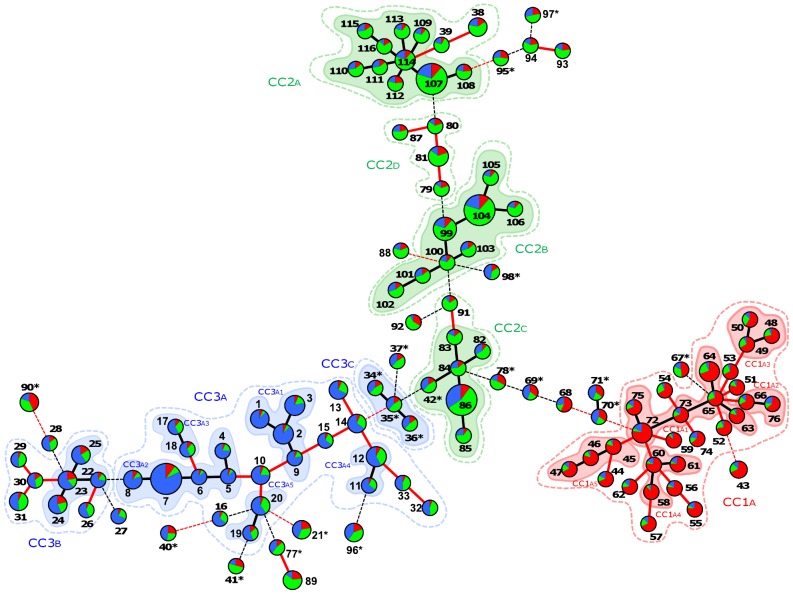
MS tree representation of clonal relationships between STs as inferred from goeBurst. Each ST is represented by a circle sized in proportion to the number of strains represented by that ST. Circles are filled with colors corresponding to the *Q* ancestry proportion as inferred by the linkage model of Structure. STs with admixture are indicated with an asterisk. Links between STs are colored according to the number of locus variations: SLV (black, thick), DLV (red, thick), TLV (black, dotted), and more than TLV (red, dotted). Clonal complexes are represented by colored shaded areas (defined by stringent grouping criteria; SLV level) and by colored dotted lines (defined by relaxed grouping criteria; DLV level).

To further investigate the differences between the three lineages and evaluate intra-lineage structure, we used goeBurst [27], an allele-based analysis optimized from the eBurst algorithm [28]. Using MLST data, goeBurst clusters STs into clonal complexes (CCs) based on the similarity of their allelic profiles. The grouping criterion can be either stringent, allowing intra-CC variation at only one of the eight loci analyzed (single locus variant; SLV) or more relaxed, allowing intra-CC variation at two loci (double locus variant; DLV). The program then facilitates the visualization of the CC pattern of descent by creating a minimum spanning (MS) tree (Figure 2). Under stringent SLV conditions, STs from lineage 1 formed five small CCs (CC1a1 to CC1a5). However, the pattern of descent of these CCs did not correspond to the positions of their member STs in the NJ tree; for example, ST72–76 formed a small monophyletic cluster in the NJ tree (Figure 1) but were connected to other STs in the MS tree. Furthermore, using the relaxed (DLV) group definition, all STs in lineage 1 formed a single large CC (named CC1a). In fact, the star-like descent pattern shown in Figure 2 is only one of several possible representations of this lineage, as it is possible to connect almost any ST from lineage 1 to any other via various DLV connection paths. For example, under DLV conditions, ST48 (CC1a3) connected to ST50, ST51, ST52, and ST54 (all in CC1a1), while ST58 (CC1a4) connected to ST63 and ST64 (CC1a1). Therefore, when considering DLV connections within lineage 1, the descent pattern for this lineage should be viewed not as a fixed path, but rather as a network of STs with short allele-sharing distances.

However, the population structure of lineage 2 was revealed to be markedly different from that of lineage 1. When we analyzed lineage 2 using stringent SLV criteria, we identified three major clonal complexes with similar population sizes (CC2a, CC2b, and CC2c). However, in contrast to lineage 1, we found that changing our grouping criterion from SLV to DLV made little difference to the overall population structure. Under relaxed DLV conditions, CC2b was unchanged, CC2c expanded to include ST91, and CC2a grouped with ST38 and ST39. We also found that the three major CCs of lineage 2 were characterized by one or two highly redundant STs, a classic feature of clonal populations. A fourth clonal complex, CC2d, formed only under the relaxed conditions created by linking DLVs. Interestingly, CC2c occupied a founder position in the MS tree and connected to both lineage 1 and lineage 3 at the triple locus variant (TLV) level.

Lineage 3 was structured in part by patterns evident in lineage 1 and in part by those observed in lineage 2. Using the relaxed group definition, this lineage contained three CCs with markedly different population sizes. The largest, CC3a, was made up of several small SLV-defined CCs (CC3a1 to CC3a5) but, unlike CC1a of lineage 1, was not structured as a network with several possible representations. The second-largest CC, CC3b, whose member STs were characterized by a lower proportion of ancestry from lineage 3 than those belonging to CC3a, weakly grouped as a clonal complex under SLV conditions. Finally, within the small CC3c, the STs (ST34–37) showed an admixture of lineage origins and linked under TLV conditions with lineage 2 through a connection to another admixed ST in CC2c (ST42).

**Figure 3:**
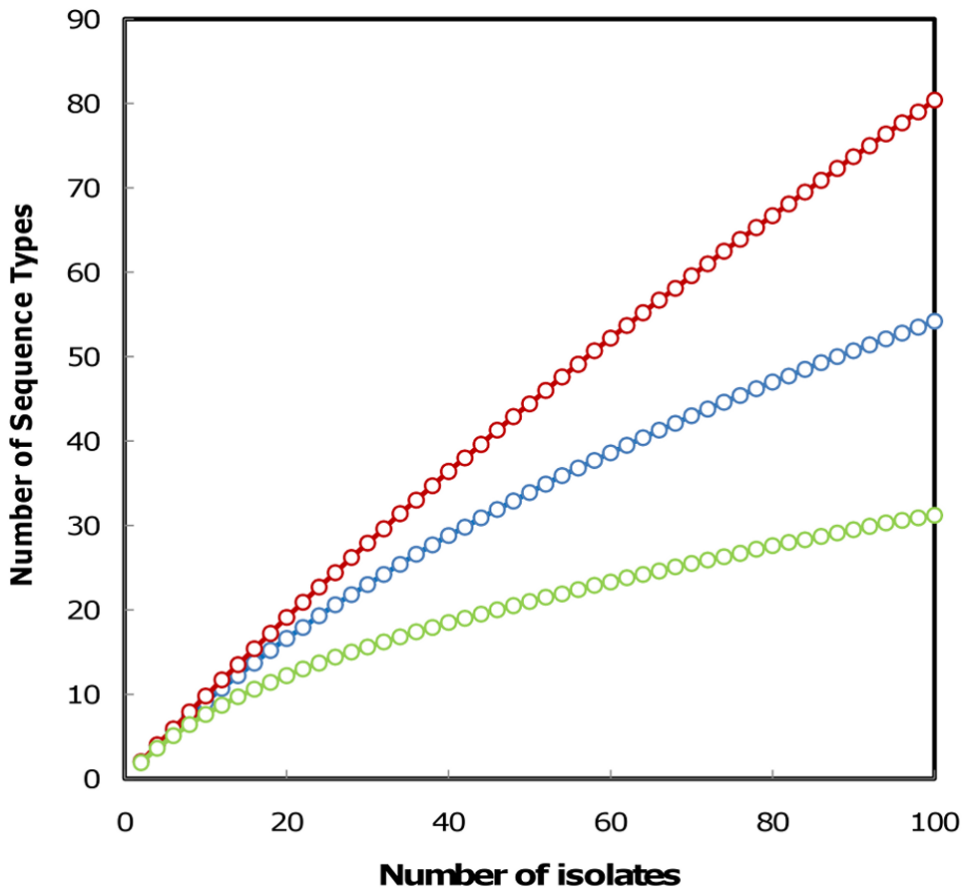
Lineage-dependent rarefaction curves. Estimated ST richness obtained as a function of the number of strains sampled, in red (lineage 1), green (lineage 2), and blue (lineage 3).

As might have been expected, most STs with admixture status did not form a structured complex. Most of these STs were connected to various CCs but linkages were weak, at three- or four-locus variant levels. Under these conditions, other grouping solutions became possible since ST94 and ST95 were four-locus variants of ST96, which was itself connected by a TLV link to lineage 3. Therefore, these data confirmed that STs with admixture status were singletons rather than a structured population of isolates. We also noted that, in lineage 1, 38 strains were sufficient to produce 30 different STs, whereas lineage 2 and lineage 3 showed a greater redundancy of STs in sampling. The rarefaction curve (Figure 3) demonstrates that genetic diversity was easily recovered in lineage 1, while lineage 2 showed a high ST redundancy in strain sampling, a probable sign of strong clonality.

### Recombination rates vary among the 3 lineages

**Figure 4:**
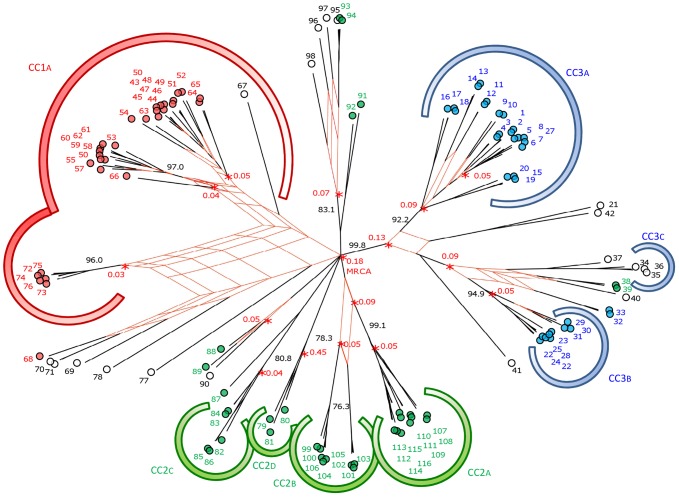
Influence of recombination on the *L. sakei* genealogy. SplitsTree consensus network based on 10,000 phylograms produced by the ClonalFrame analysis of the concatenated sequences of the 116 STs, with correction for recombination. The network of phylogenetic ambiguities was drawn for all branches having more than 20% uncertainty. Each circle represents a unique ST (labeled with ST designation) and is colored according to its lineage affiliation as inferred by Structure: red (lineage 1), green (lineage 2), and blue (lineage 3). STs with admixture are represented by open circles. Major CCs are distinguished by large colored arcs. Posterior probability values for main branches are depicted in black to the side of the branch. Time in coalescent units (based on ClonalFrame 50% majority-rule consensus tree; Figure S2) is given (in red) for the main nodes of the tree.

To further investigate the differences between the three lineages, we used ClonalFrame [29] to infer phylogeny under the neutral coalescent model and to analyze the role recombination has played in creating *L. sakei*'s population structure. We used the fifty-percent majority rule consensus tree created in ClonalFrame (available in Figure S2) as the basis for a consensus network tree (Figure 4) produced using SplitsTree 4.1 [30]. We found clear evidence of conflicting phylogenetic signals and the influence of recombination throughout the population structure, specifically in a branch of lineage 3 that contained CC3c, a branch of lineage 2 that contained admixed STs (ST95, ST96, ST97, ST98), and throughout lineage 1. The ClonalFrame genealogy also displayed branching patterns quite different from those of the NJ tree (Figure 1). Indeed, incorporating recombination into the analysis resulted in a tree that indicates that most STs in lineage 1 and 2 do not share a most recent common ancestor (MRCA) any earlier than the root of the tree at 0.18 coalescent units, suggesting that lineage 1 and lineage 2 separated rapidly into independent populations following the split with lineage 3. When we looked only at lineage 2, we found the genealogy revealed a complex structure: the different CCs from lineage 2 vary in age but appear along parallel paths emerging from the root of the tree. Their origin is thus located deep in the history of lineage 2, suggesting a strong ancestral diversification into distinct clonal populations. Only CC2a and CC2b have diverged more recently, as their MRCA appears at 0.09 coalescent units. Lineage 3 appears to have two main branches that correspond to CC3a and a branch common to CC3b and CC3c. The unique composition of the pseudo-branch that contains CC3c (ST34–36) suggests that recombination events were influential in this group's divergence from CC3b: together with the members of CC3c (ST34–36), we found admixed singleton STs (ST37 and ST40), additional STs from lineage 3 (ST32, ST33), and two STs from lineage 2 (ST38, ST39). All STs from lineage 3 share a MRCA at 0.13 coalescent units, indicating that lineage 3 is the earliest diverging branch in the genealogy.

ClonalFrame was then used to evaluate and compare recombination rates among lineages (Table 3). We found the highest relative occurrence of recombination compared to point mutation in lineage 1 ((*ρ/θ* = 1.33); it was about 1.8 times higher than that in lineage 3 (*ρ/θ* = 0.74) and 5 times higher than that in lineage 2 (*ρ/θ* = 0.25). The relative impact of recombination vs. mutation in generating genetic diversity was about 2.3 times higher in lineage 1 (*r/m = *3.18) than in lineage 2 (*r/m = *1.37). Finally, when we considered the entire population of 116 STs, we found that the relative impact of recombination in the group as a whole was higher than that in any of the individual lineages alone (*r/m = *4.95), providing evidence for the importance of inter-lineage admixture.

**Table 3:** Recombination rates inferred by ClonalFrame on separate lineages.

Lineage	Number of ST's*	Fixed value of *θ*	*ρ*	*r/m*	*ρ/θ*
Lineage 1	30	18.0	23.80 (CI: 12.33–40.02)	3.18 (CI: 1.73–5.16)	1.33 (CI: 0.67–2.23)
Lineage 2	31	22.5	5.71 (CI: 2.62–10.52)	1.37 (CI: 0.65–2.48)	0.25 (CI: 0.11–0.46)
Lineage 3	32	21.5	15.87 (CI: 8.23–26.25)	2.61 (CI: 1.40–4.17)	0.74 (CI: 0.38–1.18)
All STs	116	30.0	46.20 (CI: 33.57–62.40)	4.73 (CI: 3.53–6.23)	1.54 (CI: 1.12–2.08)

All values are the mean of 5 independent runs with their 95% credibility interval (CI) given in brackets.

* : STs with admixture were removed from the lineage-specific analysis but were present for the global analysis of all STs. For lineage 2, STs with ambiguous phylogenetic position (ST93, ST94, ST38 and ST39) were also removed.

*θ*: fixed mutation rate based on the Watterson moment estimator *θ_w_*.

*ρ*: recombination rate.

*r/m:* relative impact of recombination versus mutation in generating genetic diversity.

*ρ/θ*: relative frequency of occurrence of recombination in comparison to point mutation.

### Lineages do not correlate with food-type origin

We did not detect any strong patterns corresponding to the geographical origin (laboratory of collection) or food-type origin of strains within the three lineages (data not shown). A closer look at the largest STs associated with each of the three major CCs of lineage 2 (ST86, ST104, and ST107, respectively) revealed a broad diversity of strain origin within each ST, indicating that sampling bias did not play a significant role in the formation of the CCs. Furthermore, we noticed that the strains MFPB17D07-01 (ST50), MFPB17D07-03 (ST23), and MFPB17D07-07 (ST106), previously identified from a single slice of beef carpaccio [31], were each affiliated with a different lineage.

### Lineage 1 strains have a larger chromosome than those of lineage 2

**Figure 5:**
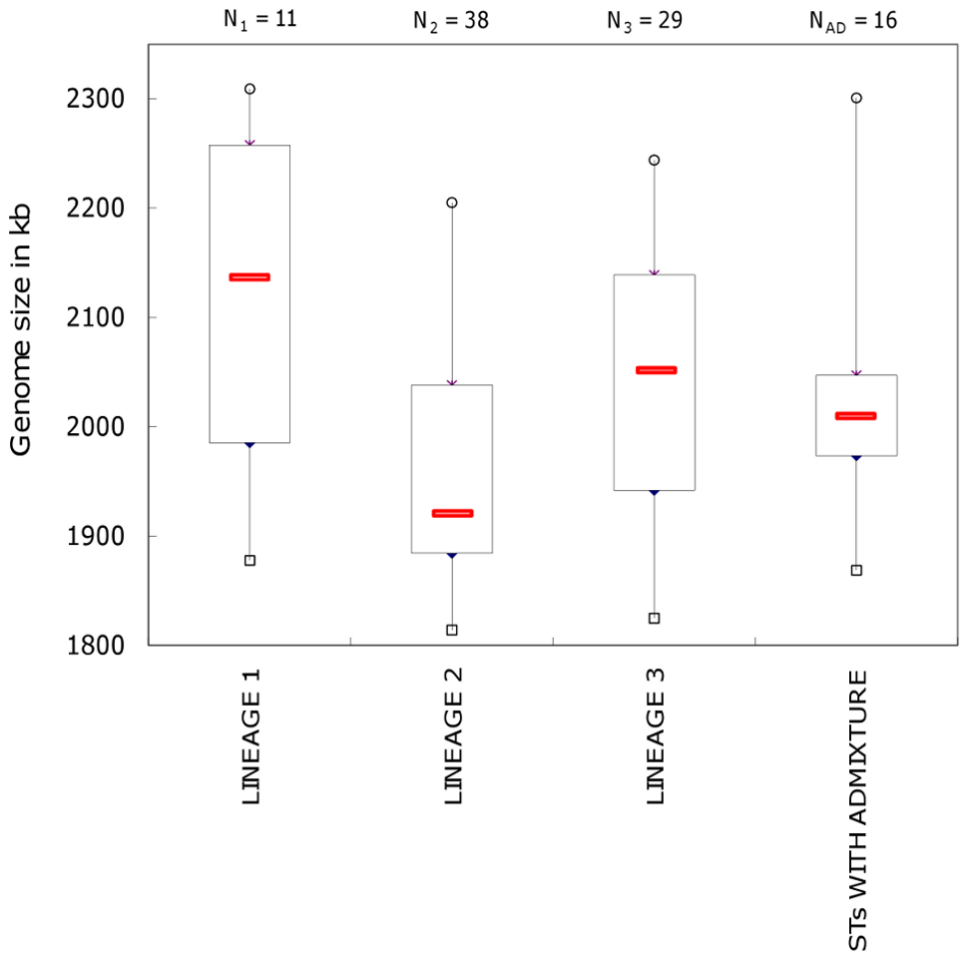
Lineage-dependent genome size of *L. sakei* strains. Box plot showing the distribution of the estimated genome size of isolates within each lineage. The median value is indicated by a red rectangle; top and bottom of boxes indicate first and third quartiles, respectively. Minimum and maximum values are shown with small rectangles and circles, respectively. The number of strains per lineage for which the genome size has been determined via pulse-field gel electrophoresis and *I*-*Ceu*I digestion is indicated above each plot (N_1_, N_2_, etc.).

However, we found significant differences among the lineages with respect to the average chromosome size of isolates (as determined with pulse-field gel electrophoresis; results shown in Figure 5). In particular, isolates from lineage 1 had a significantly larger median chromosome size than those from lineage 2 (2,147 kb vs. 1,913 kb, respectively), and a two-tail Fisher's exact test rejected the null hypothesis of independence between genome size and affiliation to lineages 1 and 2 (*p* = 0.037). Isolates from lineage 3 had a median chromosome size intermediate to those from lineages 1 and 2 (2,053 kb), which was not significantly different from either of the other two lineages.

## Discussion

In this report, we used MLST to analyze the SNPs and allelic distribution of eight loci within a *L. sakei* population of 232 strains. Our analysis demonstrated that this species has a complex population structure and revealed a pattern of diversity different from those previously published [8], [10], [11]. Through the implementation of various analytical methods, we have established that the contemporary population of strains is derived from three ancestral lineages, each with a unique population structure.

### Lineage 1 is a panmictic subpopulation

Several lines of evidence support this interpretation. First, we observed that this lineage was highly diverse, as the number of strains per ST was close to 1 and the slope of the rarefaction curve of ST accumulation as a function of increased sampling effort did not approach zero. Second, the goeBurst analysis showed a random distribution of alleles within this lineage indicative of a reticulate structure. Third, the relative impact of homologous recombination vs. mutation in generating lineage 1 genetic diversity (*r/m = *3.18) puts lineage 1 on par with species with high recombination rates, such as *Campylobacter jejuni* [32]. In fact, taken together, these three observations suggest a high occurrence of intra-lineage recombination in lineage 1, leading most likely to strong intra-lineage homogenization and to poorly-delineated, “fuzzy” subpopulations. We also found strong evidence for the importance of recombination in generating lineage 1 diversity in the observation that the average genome size of lineage 1 isolates was ∼200 kb larger than that of isolates from lineage 2, a lineage in which the influence of recombination is much less obvious. It is likely that horizontal gene transfer has played an important role in this lineage across evolutionary time and has led to an increase in the genomic content. It should be noted that lineage 1 shares very few alleles with lineage 2 and 3, suggesting that it is evolving along an evolutionary path distinct from those of the two other lineages.

### Lineage 2 is a clonal, specialized subpopulation

In contrast to lineage 1, there is significant evidence of clonality in lineage 2. Lineage 2 is composed of three major CCs that each contains a highly abundant founder ST. Within lineage 2, recombination has played less a role in generating genetic diversity than in lineages 1 and 3 (*r/m = *1.37). Unfortunately, it was not possible to unambiguously elucidate the phylogenetic relationships between the CCs of lineage 2, either because evidence of the ancestral population has been erased as a result of purifying selection, or because the intermediate diversity of lineage 2 may have not been captured properly during our sampling. Thus, the genealogical relationships deep within lineage 2 remain largely unresolved. However, some of our observations suggest that the role of clonality in creating lineage 2's population structure may have changed gradually over time. In particular, CC2c shows more allelic similarity to lineages 1 and 3 than to the other CCs of lineage 2, placing it in a central position in the pattern of evolutionary descent in the MS tree. Also, CC2a and CC2b have emerged more recently (∼0.09 coalescent units) and the genotypic (ST) diversity of these two CCs is currently expanding rapidly. From our data, we could not clearly establish whether or not these two CCs evolved from CC2c, and similarly, the position of CC2d remained quite ambiguous since the goeBurst and ClonalFrame analyses delivered conflicting results. Finally, isolates from lineage 2 had the lowest average genome size within the overall *L. sakei* population, indicating that clonal expansion might have been associated in this case with a slight reduction of genome content, a classic feature of ecological specialization.

### Lineage 3 is a blend of evolutionary paths

The genealogy of lineage 3 was more easily resolved than those of lineages 1 and 2 by the ClonalFrame analysis. All lineage 3 STs share a MRCA at 0.13 coalescent units, which represents the earliest branch in the genealogy. Lineage 3 thus appears to have conserved more intermediate diversity than the other lineages and has evolved under the nearly equally balanced influences of recombination and mutation. In fact, we found evidence of several different phylogenetic signals within lineage 3: in particular, the branch containing CC3a shows evidence of clonality, whereas the branch common to CC3b and CC3c contains more phylogenetic ambiguities. The CC3b/CC3c branch is composed in large part of admixed STs, and thus represents a phylogroup marked by the influence of recombination.

### Admixture appears mainly between lineage 2 and lineage 3

STs characterized by admixture represented about 10% of the entire population of strains. These hybrid STs may constitute a pseudo-population in the process of divergence, or, alternatively, they may merely be the result of independent recombination events in individuals with an ephemeral ecological life-span. With the exception of the few STs that appeared to be the result of complete admixture of the three lineages (e.g. ST90 and ST96), our data showed that the hybrid population was mainly composed of hybrids between lineages 2 and 3. The identification of more admixed strains is required in order to be able to draw definite conclusions regarding the emergence of a new subpopulation.

### Lineages arose from ecological adaptive divergence

The phylogenetic topology inferred by ClonalFrame and the net nucleotide distance inferred by Structure suggest that a first speciation event separated lineage 3 from another ancestral lineage, which then further split into lineages 1 and 2. However, this scenario is not supported by our observation that most of the hybrid STs we found were genetic mixtures of lineages 2 and 3; furthermore, these two populations show a tendency to be clonal. Our understanding of the relationships between the lineages is further clouded by the observation that lineage 1 seems to have diverged from lineages 2 and 3 in terms of genomic content. Therefore, the pattern of evolutionary descent between the three lineages remains unresolved.

In the case of *L. sakei* and other bacteria found worldwide in food products originating from domesticated animals, speciation through geographical isolation appears to be unlikely. Instead, the ecological specialization may provide a more convincing explanation of the emergence of the distinct evolutionary paths of the three lineages. Indeed, ecological processes have been observed to cause a given bacterial population to evolve as either a recombining “fuzzy” population or a highly genetically coherent clonal population [33]. This evolutionary scenario could also explain the different recombination rates observed among the three lineages. For example, if occupation of a given niche is associated with a higher level of species complexity, more genetic diversity would be involved in each recombination event, thus influencing the measurement of a lineage's recombination rate. The frequency of recombination in a given lineage could also be affected by its degree of niche specialization, as we might expect to more easily detect recombination in lineages occupying a larger number of niches. Therefore, it is likely that the three lineages reflect adaptations of *L. sakei* to three different environmental/food reservoirs. Such a situation has already been observed in strains of the wine-making species *O. oeni*, in which two subpopulations differed from each other based partially on the type of fruits/grapes from which they were isolated [34].

Specifically identifying the *L. sakei* reservoirs remains a difficult task. Each of the three lineages identified in the current study contained isolates found across the globe and at all levels of the meat food chain, a result that is consistent with previous findings [11]. We also found here that three different isolates, each a member of a different lineage, were identified on the same meat slice, highlighting the challenge of identifying lineage niche specialization. *L. sakei* is most likely a bacterium that lives in agricultural areas [11], [35], [36], a habitat that contains several ecological niches (spatial and temporal) to which the species may have adapted, including pasture and barn environments, water fowl, silage, and the intestinal tracts of animals. Thus, the different evolutionary histories of *L. sakei* lineages might reflect adaptation to some of these microhabitats. Livestock domestication and food processing may also play an important role in selecting particular behaviors or evolutionary strategies, as has been demonstrated for three species of paramount importance to the dairy industry, *Streptococcus thermophilus*, *Lactococcus lactis*, and *Lactobacillus casei.* These species all demonstrate clonal expansion, which is likely correlated with human domestication of bovids and subsequent development of milk fermentation processes [15], [21], [22]. The situation may be different for meat or fish products, but modern meat processing techniques are known to exert strong selective pressures on bacterial development [31], [35]. Therefore, there might be a possible role of the food chain in selecting particular ecotype (*i.e*. clonal expansion)”. More population-level analyses are needed to test these hypotheses, including a larger environmental sampling strategy and the development of tools to follow *L. sakei*'s subpopulation dynamics in food products.

### Our data are updating those of previous reports

The MLST results presented here confirm in part the results of a previous study based on a PCR binary typing method [11]. The three phylogenetic lineages described here represent a set of about 8 to 10 phylogroups (depending on whether or not admixed STs are included), which corroborates our previous identification of about 10 different genotypic clusters within this species. The genotypic positions of the 73 strains analyzed in both studies indicate a strong correlation of lineage 1 with clusters I and J (nomenclature taken from [11]) and lineage 2 with clusters A and C (see Table S1). However, genotypic clusters from [11] that contained admixed strains and strains from lineage 3 did not correspond to the CCs identified in our MLST study. This lack of correspondence in the results for lineage 3 could potentially stem from methodological differences between the two studies, as the PCR binary typing method was based mainly on genes of strain 23K from lineage 2. Therefore, our genotyping protocol could be improved in future analyses by adding gene markers identified in strains from lineages 1 and 3.

Finally, the division that we report here of the *L. sakei* population into three lineages is inconsistent with the current separation of the species into two subspecies [8]. As already discussed in our previous report [11], subdividing a species based on variation in a single abundant protein (GapA) (as per protein electrophoresis patterns) has clear limitations when attempting to assess the real diversity of genotypes within *L. sakei*. Furthermore, the use of a single gene marker in a population of strains in which the influence of recombination is rather high renders the results of such an analysis unreliable and has the potential to lead to erroneous conclusions. In addition, modern identification of most *L. sakei* strains isolated from food does not involve protein profile pattern analysis. We thus suggest that this biochemical test should no longer be used to assign strains to two “subspecies”. We also propose that the subspecies definition should be abandoned or revised and be replaced by the lineages defined here.

## Conclusion

The present study provides a description of *L. sakei* intraspecific diversity that is supported by multiple analytical methods. We have shed light on how this species has evolved into three separate lineages, each with a different population structure. These findings should prompt new lines of research that evaluate and measure the possible ecological differences underlying the three lineages. Furthermore, the MLST scheme presented here represents an easy and straightforward tool that, together with the construction of a public database, should aid in the further analysis of the population dynamics of *L. sakei* strains in food products.

## Materials and Methods

### Bacterial strains

The *L. sakei* 232 strains analyzed in this study are listed in Table S1 provided as supporting information. They were collected from 19 laboratory collections [6], [9]–[11], [31], [37]–[48] out of 12 countries throughout the five continents. The strains were collected from various types of food, fermented or not (meat, seafood, vegetables), and human feces. The strains were selected on prior knowledge of their phenotypic, genotypic or geographic diversity. Strains from Human feces were isolated previously and anonymously with compliance to ethic statements as described in [39], [42]. This collection of strains was considered as being representative of the species diversity in meat or fish products. Bacteria were grown overnight at 30°C on MRS broth (Oxoid, Cambridge, UK). Total DNA was extracted from 200 µl of culture using the High Pure PCR Template preparation kit (Roche Diagnostics, Basel, Switzerland) according to the manufacturer's instructions.

### Design of MLST scheme

The *L. sakei* MLST scheme was elaborated from an initial list of 50 genes already used for other bacterial MLST analysis (http://pubmlst.org/) and for which homologous sequences were identified in the *L. sakei* 23K genome. These 50 loci were chosen to represent a wide range of positions through the *L. sakei* 23K chromosome, and to cover a diversity of biological functions. This analysis resulted in a pre-selection of 16 genes: *ddl* (D-alanine/D-alanine ligase; LSA0049), *guaA* (guanosine monophosphate synthase; LSA0139), *mutL* (DNA mismatch repair protein; LSA0363), *pepV* (carnosinase; LSA0424), *recA* (DNA recombinase A; LSA0487), *glpF* (glycerol facilitator; LSA0651), *ftsQ* (cell division protein; LSA0749), *nrdE* (ribonucleoside-diphosphate reductase, alpha chain; LSA0941), *tuf* (elongation factor Tu; LSA1043), *dnaK* (chaperone protein; LSA1236), *hemN* (coproporphyrinogen oxidase; LSA1240), *glnA* (glutamine synthetase; LSA1321), *nadC* (nicotinate phosphoribosyltransferase; LSA1570), *ldhL* (L-lactate dihydrogenase; LSA1606), *alr* (alanine racemase; LSA1613) and *rpoB* (RNA polymerase, beta subunit; LSA1775). The entire sequence of the 16 loci was determined on a selection of 20 strains (23K, CIP105422, ATCC15521, 300, G3, 156, Lb706, 160*1, CTC163, SF841, MF2090, MF2092, LTH5589, LTH673, 112, 332, 64, LTH677, LTH675, BMG.136) affiliated to the 10 genotypic clusters previously described [11]. The most polymorphic internal region of each locus was chosen according to its capacity to measure intra-species diversity through the estimation of polymorphic sites, number and distribution of alleles. Upon these criteria, one gene, *nrdE* was removed because a plasmidic paralog was found to be present in some strains. Three other genes, *glnA*, *nadC* and *ftsQ* showed a very low level of polymorphism. At last, 4 genes *ddl*, *mutL*, *guaA* and *alr* showed significant traces of intragenic recombination. Therefore, the 8 following genes (*pepV*, *recA*, *glpF*, *tuf*, *dnaK*, *hemN*, *ldhL*, *rpoB*) were selected for the MLST scheme.

### Primers, PCR assay and DNA sequencing

Primers were designed from the sequence of the eight loci from strain 23K and are listed in Table S2. PCR amplifications were done with proofreading Pwo enzyme (Roche Diagnostics, Basel, Switzerland) and were performed in 50-µl reactions containing 10 ng of total genomic DNA, 200 µM of each dNTPs, 200 nM of forward/reverse primers at 1.5 mM MgSO_4_ concentration. PCR conditions started with an initial denaturation step of 4 min at 95°C, followed by 30 cycles of 30 s denaturation at 95°C, 1 min annealing at 55°C, 1 min elongation at 72°C. PCR products were individually checked after agarose gel electrophoresis migration and subsequently cleaned from residual primers using QIAquick PCR purification kit (Qiagen, Courtaboeuf, France). PCR product were then sequenced by Sanger sequencing service (Eurofins MWG operon, Eberberg, Germany) using big-dye terminator chemistry. The quality of sequences was checked in Consed editor after assembly of all traces per loci with Phred/Phrap software suite [49]. Sequencing was repeated if a polymorphic site occurred in only one strain. Genome size estimation were taken from already available data [11], [50], but were also determined for new strains of a recent sampling in Tunisian food products [43] by pulse-field gel electrophoresis and *I-Ceu*I digestion performed as described earlier [11].

### Descriptive analysis of sequence data

Assignment of alleles and of ST numbers, nucleotide diversity (π, average pairwise nucleotide differences/site), the number of polymorphic sites (S), G+C content, The Tajima's *D* test for neutrality were calculated with DnaSP software version 5.0 [51]. Concatenated sequences and haplotypes representative sequences were generated with custom Perl script developed on a Linux Bioinformatic Plateform at INRA Jouy-en-Josas (http://migale.jouy.inra.fr/). Rarefaction curves were calculated using Resampling Rarefaction software version 1.3 (http://strata.uga.edu/software/index.html).

### Population analysis

The Bayesian approach implemented in Structure software version 2.3 [24], [25] was used to infer the lineage ancestry of the 116 unique STs assuming that each ST has derived all of its ancestry from *K* ancestral subpopulations. The number of population *K* was determined under the linkage model. *K* was estimated by comparing *P*(X|*K*), the posterior probability that the X genotypes of the sampled individuals are belonging to the assumed *K* populations (see Figure S3). Twelve individuals runs per value of *K* (chosen between 2 and 10) were performed using 100,000 burn-in iterations and 200,000 sampling iterations. The *K* value that generated the highest median posterior probability was used as the number of possible population. To compare the *K* ancestral populations, several model parameters were analysed from the Structure output file such as the net nucleotide distance among the *K* population, the average distance between STs in the same population and *Q* the multi-dimentional vector of ancestry proportions for all STs.

Global Optimal eBurst analysis (http://goeBURST.phyloviz.net) was carried out to cluster the 116 STs to clonal complexes based on their allelic profiles (with respect to their number of SLVs, DLVs or TLVs) and to further infer an hypothetical pattern of evolutionary descent by constructing a MS Tree from the eBurst data [27].

### Phylogenetic analysis & recombination

Phylogenetic analysis was carried out using the neighbor-joining method with a Kimura two-parameters distance model implemented in MEGA software version 4.0 [52]. Bootstrap values were obtained after 1,000 replicates. The RETICULATE program [25] implemented in RDP3 software [53] was used to evaluate the phylogenetic concordance between each parsimony informative polymorphic sites. Phylogenetic and evolutionary relationships between individual ST were inferred using ClonalFrame software version 1.1 [29]. The genealogy of the population and the time, in coalescent unit, to the most recent common ancestor were measured with correction for recombination occurring in the bacterial population. The sequence data of the 116 unique STs were used as input data. Five independent runs were performed for each analysis, with a burn-in cycle of the MCMC (Markov Chain Monte Carlo) algorithm fixed to 100,000 iterations and a posterior sampling of 200,000 iterations. The prior iterations were discarded and model parameters were sampled in the second period of the run at every 100 iterations, resulting in 2,000 samples from the posterior. Satisfactory convergence of the MCMC in the different runs was estimated based on the Gelman-Rubin statistic implemented in ClonalFrame. The genealogy of the population was summarized and the robustness of the tree topology was evaluated by concatenating the posterior samples of the five runs to built-up a 50% majority rule consensus tree using the ClonalFrame GUI, and the consensus network algorithm implemented in SplitsTree 4.1 [30]. For the evaluation of the relative impact of the mutation rate (*θ*) and of the recombination rate (*ρ*) on the whole population of 116 STs and within specific lineages, a similar fixed value of δ = 406 bp (δ = mean tract length of imported sequence fragment) being the value inferred during the five runs carried out on the whole dataset. This value of δ was closely related to the average fragment length sequenced for the eight loci (420 bp). We also fixed the mutation rate to the Watterson's moment estimator *θ*_W_ [54] obtained from the whole population or from each lineage population from which STs with admixture were discarded. Five runs were also carried out during this lineage-specific analysis but a burn-in period of 200,000 iterations was required for the convergence of the MCMC. From these runs, several measures were also taken such as *ρ/θ* (relative frequency of occurrence of recombination and mutation), r/m (relative impact of recombination and mutation in the diversification of the lineages).

### Nucleotide sequence accession numbers

Nucleotide sequences have been deposited in GenBank under accession numbers KC771901 to KC772132 (*pepV*), KC772133 to KC772364 (*recA*), KC772365 to KC772596 (*glpF*), KC772597 to KC772828 (*tuf*), KC772829 to KC773060 (*dnaK*), KC773061 to KC773292 (*hemN*), KC773293 to KC773524 (*ldhL*) and KC773525 to KC773756 (*rpoB*).

## Supporting Information

Figure S1 **Compatibility matrix for nucleotide polymorphisms within and between loci using Reticulate program.** The matrix contains all pairwise comparisons of 137 binary parcimony informative sites that are phylogenetically compatible (white square) or incompatible (black square). Within-locus compatibility was very high for the eight loci. In contrast, in between loci compatibility was very low and almost absent.
(PDF)

Figure S2 **Clonal genealogy inferred by ClonalFrame for the 116 unique STs.** The fifty-percent majority rule consensus tree that incorporates recombination in the phylogenetic reconstruction is presented. Branches supported by a posterior probability of more than 90% are indicated in red. STs are colored according to their lineage affiliation as inferred by Structure: red (lineage 1), green (lineage 2), and blue (lineage 3). STs with substantial admixture are kept in black.
(PDF)

Figure S3 **Estimation by Structure (using linkage model) of posterior** ***P*** **(X|** ***K*** **) likelihood variability as a function of** ***K*** **populations in the** ***L. sakei*** **population of 116 STs.** Each circle represents the output of a single Structure run (12 carried out per value of *K*). Median values are depicted by red rectangles.
(PDF)

Table S1 **List and identifying information of strains used in this study, listed according to their ST affiliation.** Allelic profiles are given. Lineage and clonal complex affiliation as inferred by Structure and goeBurst are also indicated. Finally, pulse-field electrophoresis estimation of genome size and affiliation to previously described genotypic clusters are mentioned (when available).
(PDF)

Table S2 **Description of primers used in the MLST analysis.**
(DOC)
