# Supplementary figures and images for: Population Genetics of Lactobacillus sakei Reveals Three Lineages with Distinct Evolutionary Histories
Source: PLoS One. 2013 Sep 19;8(9):e73253. doi: 10.1371/journal.pone.0073253 (PMC3777942; doi:10.1371/journal.pone.0073253)

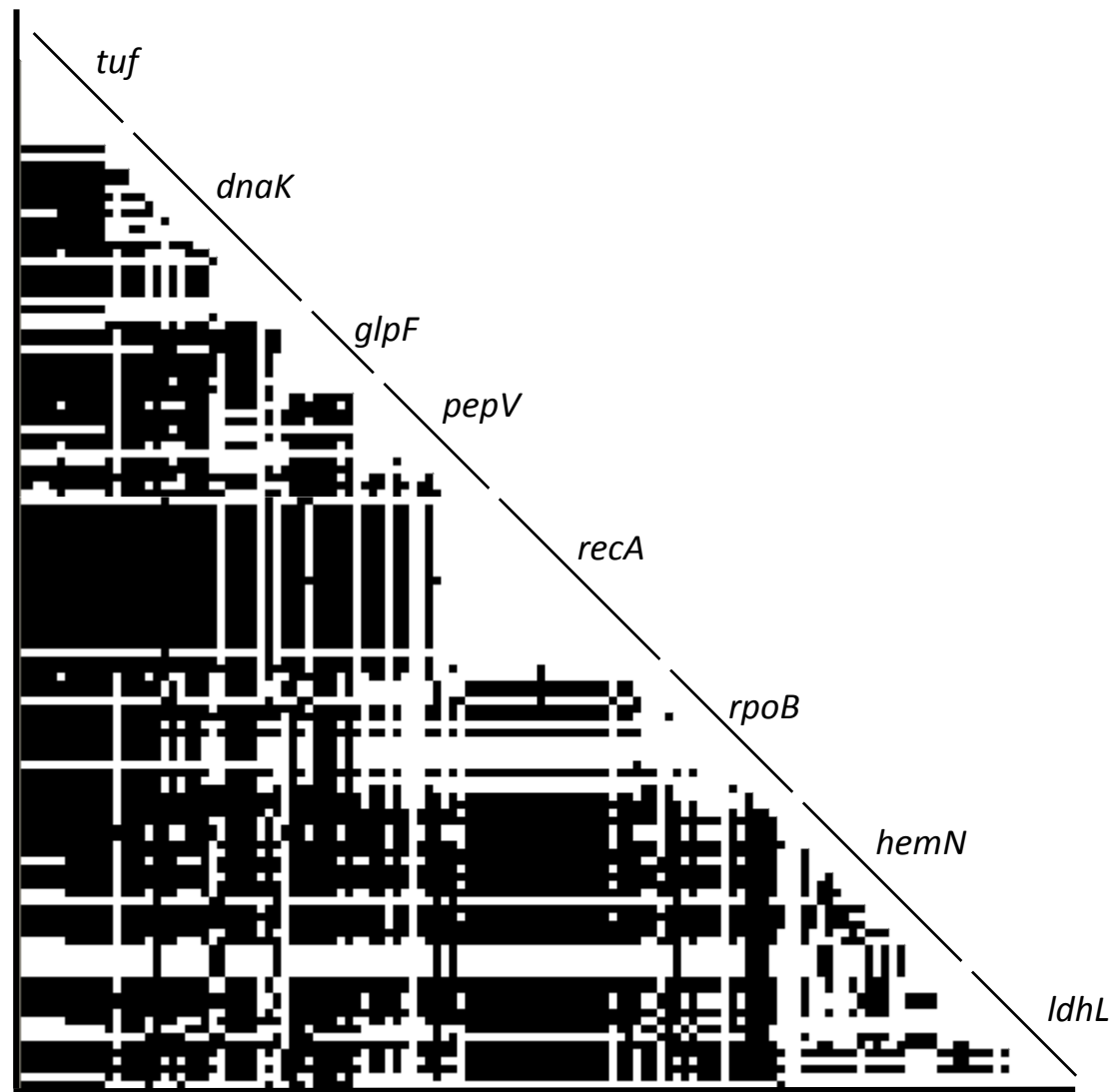

Figure S1

Supplement: Figure S1 — Compatibility matrix for nucleotide polymorphisms within and between loci using Reticulate program. The matrix contains all pairwise comparisons of 137 binary parcimony informative sites that are phylogenetically compatible (white square) or incompatible (black square). Within-locus compatibility was very high for the eight loci. In contrast, in between loci compatibility was very low and almost absent. (PDF) [file pone.0073253.s001.pdf]

Figure S2

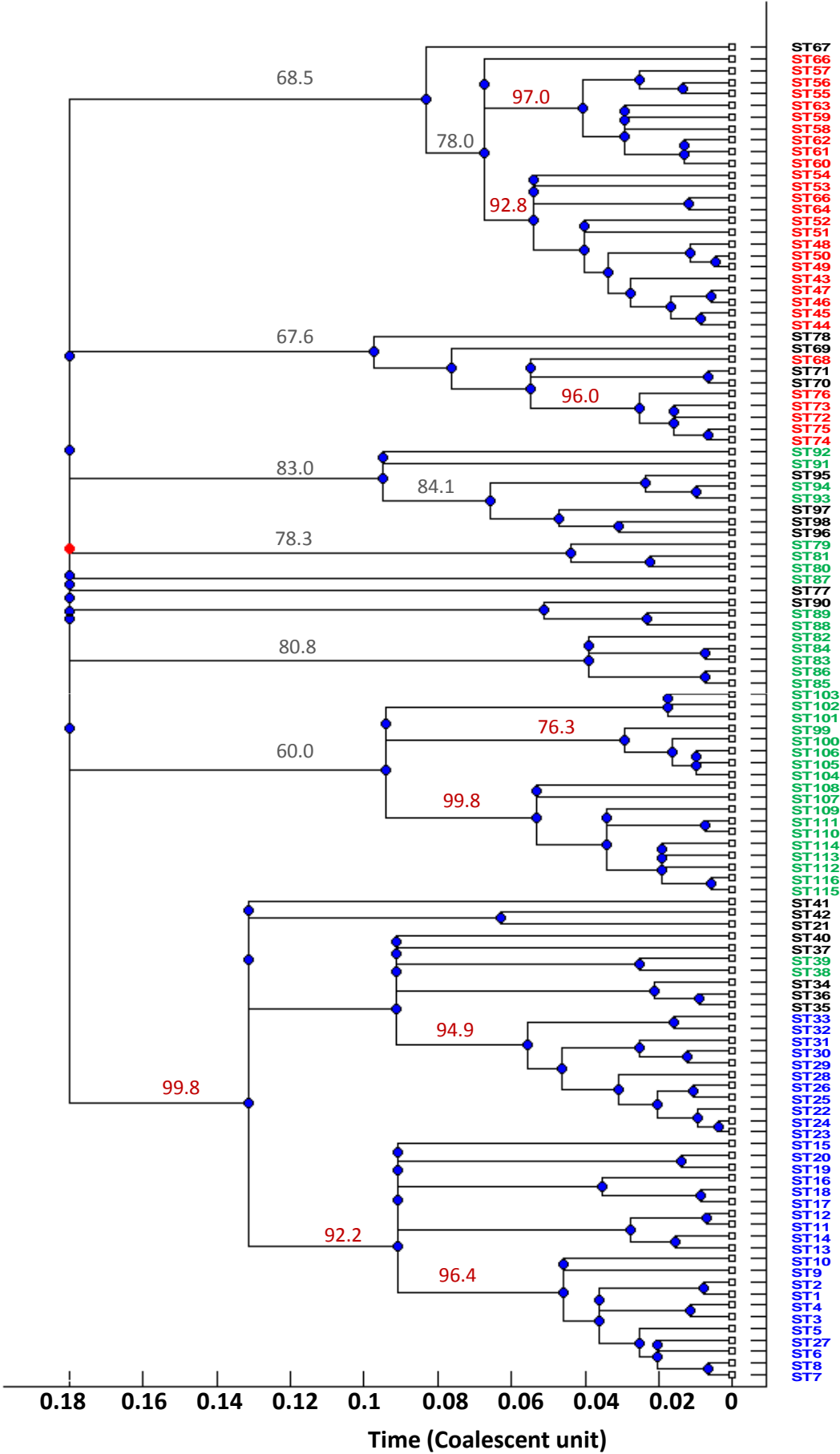

Supplement: Figure S2 — Clonal genealogy inferred by ClonalFrame for the 116 unique STs. The fifty-percent majority rule consensus tree that incorporates recombination in the phylogenetic reconstruction is presented. Branches supported by a posterior probability of more than 90% are indicated in red. STs are colored according to their lineage affiliation as inferred by Structure: red (lineage 1), green (lineage 2), and blue (lineage 3). STs with substantial admixture are kept in black. (PDF) [file pone.0073253.s002.pdf]

Figure S3

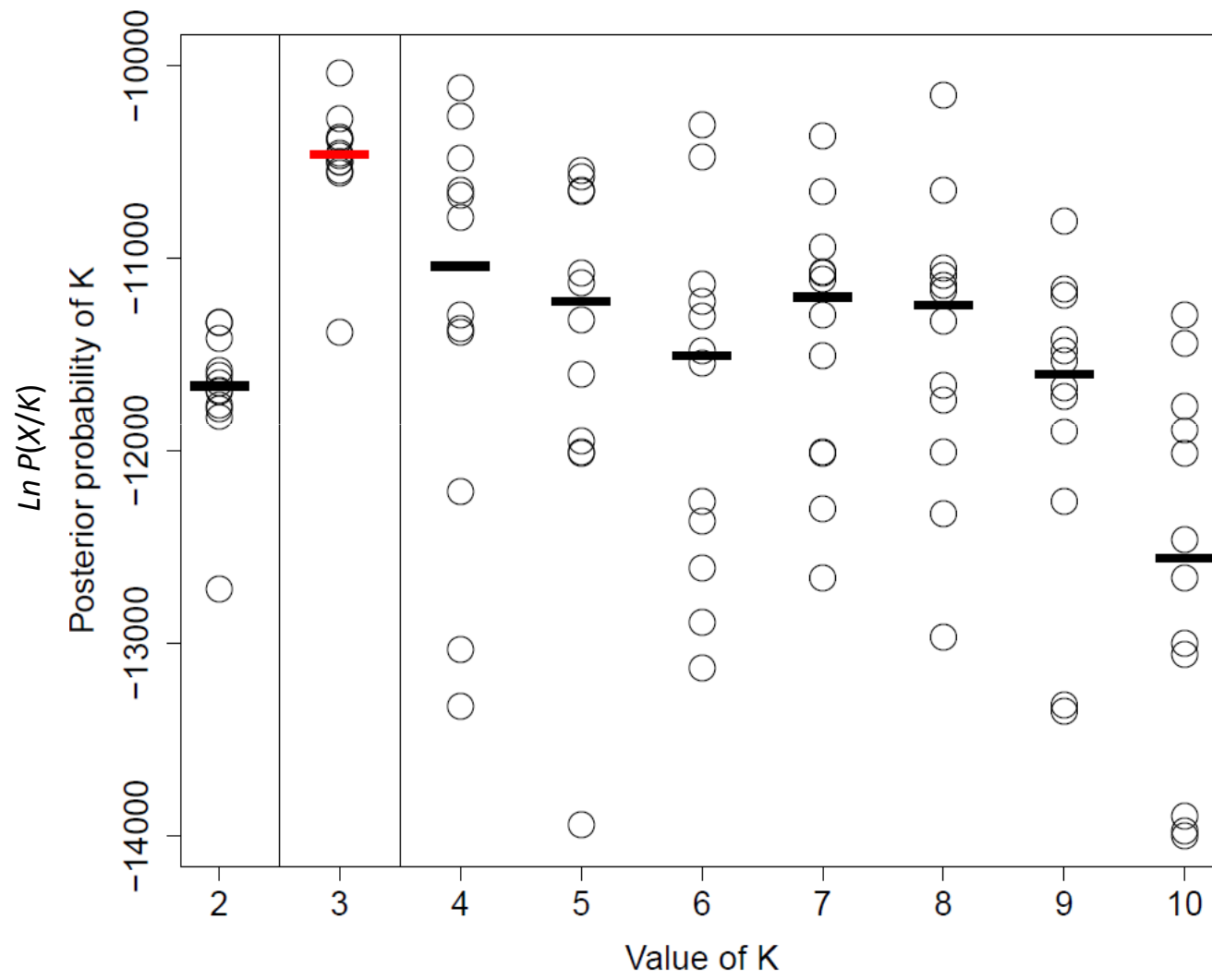

Supplement: Figure S3 — Estimation by Structure (using linkage model) of posterior P (X| K ) likelihood variability as a function of K populations in the L. sakei population of 116 STs. Each circle represents the output of a single Structure run (12 carried out per value of K). Median values are depicted by red rectangles. (PDF) [file pone.0073253.s003.pdf]
