# Supplementary material for: Population Genetics of Lactobacillus sakei Reveals Three Lineages with Distinct Evolutionary Histories
Source: PLoS One. 2013 Sep 19;8(9):e73253. doi: 10.1371/journal.pone.0073253 (PMC3777942; doi:10.1371/journal.pone.0073253)
Supplement: Table S1 — List and identifying information of strains used in this study, listed according to their ST affiliation. Allelic profiles are given. Lineage and clonal complex affiliation as inferred by Structure and goeBurst are also indicated. Finally, pulse-field electrophoresis estimation of genome size and affiliation to previously described genotypic clusters are mentioned (when available). (PDF) [file pone.0073253.s004.pdf]

Name of Isolate	Sequence Type	Lineage	Clonal Complex	Genotype [ref. 11]	Allelic profile								Isolation source	Date	Laboratory Collection	Reference	Genome in kb
					tuf	*recA*	*glpF*	*pepV*	*ldhL*	*rpoB*	*dnaK*	*hemN*					
CIP105422	ST1	3	CC3A	G	1	1	1	1	1	1	1	1	Raw German-type Sausage	1990	Institute of Meat Hygiene, Berlin, Germany	[9]	2077
CRL1757	ST1	3	CC3A		1	1	1	1	1	1	1	1	Fresh anchovies	2008	CERELA, Tucumán, Argentina	Unpublished	
112	ST2	3	CC3A	B	1	1	1	1	2	1	1	1	French dry-type sausage	1990-1992	INRA, Jouy-en-Josas, France	[10]	2152
134	ST2	3	CC3A		1	1	1	1	2	1	1	1	French dry-type sausage	1990-1992	INRA, Jouy-en-Josas, France	[10]	
129	ST2	3	CC3A		1	1	1	1	2	1	1	1	French dry-type sausage	1990-1992	INRA, Jouy-en-Josas, France	[10]	
113	ST2	3	CC3A		1	1	1	1	2	1	1	1	French dry-type sausage	1990-1992	INRA, Jouy-en-Josas, France	[10]	
FLEC01	ST3	3	CC3A		1	1	1	1	2	9	1	1	Human feces	2008	INRA, Jouy-en-Josas, France	[42]	1966
PFHL-F1	ST3	3	CC3A		1	1	1	1	2	9	1	1	Fresh Duck breast	2006	INRA, Jouy-en-Josas, France	Unpublished	
205	ST3	3	CC3A	B	1	1	1	1	2	9	1	1	Vacuum-Packed beef	1987	INRA, Jouy-en-Josas, France	[37]	1825
ADIV-DM3	ST3	3	CC3A		1	1	1	1	2	9	1	1	Traditional dry-type French Sausage	Not known	ADIV, Clermont-Ferrand, France	Unpublished	
MFPC15A28-05	ST3	3	CC3A		1	1	1	1	2	9	1	1	Vacuum Packed beef carpaccio	2009	INRA, Jouy-en-Josas, France	[31]	
MFPA29D07-01	ST3	3	CC3A		1	1	1	1	2	9	1	1	Modified-Atmosphere Packed organic beef carpaccio	2009	INRA, Jouy-en-Josas, France	[31]	
AUA-985	ST4	3	CC3A		1	1	1	1	4	9	1	2	Industrial dry-type Greek sausages	2007	Agricultural University, Athens, Greece	Unpublished	
324	ST5	3	CC3A		1	1	1	1	4	3	1	2	Vacuum-Packed pork	1987	INRA, Jouy-en-Josas, France	[37]	
331	ST6	3	CC3A	B	1	1	1	1	4	3	5	2	Vacuum-Packed pork	1987	INRA, Jouy-en-Josas, France	[37]	2208
LTH673	ST7	3	CC3A	G	1	1	1	1	4	3	5	3	German moist-type fermented sausage	1991	Institut Für Libbensmitteltechnologie, Hohenheim, Germany	[48]	2092
LV92	ST7	3	CC3A	G	1	1	1	1	4	3	5	3	Bacon	1983	Meat Research Institute, Bristol, UK	[47]	
AGR63	ST7	3	CC3A		1	1	1	1	4	3	5	3	Vacuum-packed beef	2008	AgResearch, Hamilton, New-Zealand	[6]	
SAM125	ST7	3	CC3A		1	1	1	1	4	3	5	3	Greek-type dry salami	1994	National Agricultural Research Foundation, Ioannina, Greece	[44]	
LV52	ST7	3	CC3A	G	1	1	1	1	4	3	5	3	Vacuum-packed lamb meat	1983	Meat Research Institute, Bristol, UK	[47]	2162
91	ST7	3	CC3A		1	1	1	1	4	3	5	3	French dry-type sausage	1990-1992	INRA, Jouy-en-Josas, France	[10]	
48	ST7	3	CC3A		1	1	1	1	4	3	5	3	French dry-type sausage	1990-1992	INRA, Jouy-en-Josas, France	[10]	
SAM517	ST7	3	CC3A		1	1	1	1	4	3	5	3	Greek-type dry salami	1994	National Agricultural Research Foundation, Ioannina, Greece	[44]	
LV59	ST7	3	CC3A	G	1	1	1	1	4	3	5	3	Vacuum-packed lamb meat	1983	Meat Research Institute, Bristol, UK	[47]	2029
MFPA29D07-04	ST7	3	CC3A		1	1	1	1	4	3	5	3	Modified-Atmosphere Packed organic beef carpaccio	2009	INRA, Jouy-en-Josas, France	[31]	
195	ST7	3	CC3A	G	1	1	1	1	4	3	5	3	Vacuum-Packed beef	1987	INRA, Jouy-en-Josas, France	[37]	2039
ADIV-L28	ST7	3	CC3A		1	1	1	1	4	3	5	3	Industrial dry-type French sausage	Not known	ADIV, Clermont-Ferrand, France	Unpublished	
SAM234	ST8	3	CC3A		1	1	1	2	4	3	5	3	Greek-type dry salami	1994	National Agricultural Research Foundation, Ioannina, Greece	[44]	
AGR27	ST8	3	CC3A		1	1	1	2	4	3	5	3	Vacuum-packed beef	2008	AgResearch, Hamilton, New-Zealand	[6]	
AGR44	ST9	3	CC3A		1	1	10	1	2	1	1	1	Vacuum-packed beef	2008	AgResearch, Hamilton, New-Zealand	[6]	
LTH5589	ST10	3	CC3A	C	1	1	10	1	4	1	1	2	Human feces	2002	Institut Für Libbensmitteltechnologie, Hohenheim, Germany	[39]	2142
YMH243	ST10	3	CC3A	C	1	1	10	1	4	1	1	2	Fresh meat	1986	National Institute of Health, Tokyo, Japan	[41]	1980
LTH5588	ST11	3	CC3A	B	1	1	10	2	2	9	7	2	Human feces	2002	Institut Für Libbensmitteltechnologie, Hohenheim, Germany	[39]	1909
LTH1650	ST12	3	CC3A		3	1	10	2	2	9	7	2	Starter strain for German-type sausages	not known	Institut Für Libbensmitteltechnologie, Hohenheim, Germany	Unpublished	
L110	ST12	3	CC3A	B	3	1	10	2	2	9	7	2	Starter strain for French-type sausages	1987	INRA, Jouy-en-Josas, France	[37]	1863
475	ST12	3	CC3A		3	1	10	2	2	9	7	2	Vacuum-Packed beef	1987	INRA, Jouy-en-Josas, France	[37]	
LTH1182	ST12	3	CC3A		3	1	10	2	2	9	7	2	German-type sausage	1991	Institut Für Libbensmitteltechnologie, Hohenheim, Germany	[48]	
MF2092	ST13	3	CC3A	H	5	1	10	1	1	1	7	2	Smoked Salmon	2004	NOFIMA, Åas, Norway	[11]	2059
MF1048	ST13	3	CC3A	H	5	1	10	1	1	1	7	2	Smoked Salmon	2004	NOFIMA, Åas, Norway	[11]	1942
186	ST14	3	CC3A		5	1	10	2	2	1	7	2	French dry-type sausage	1990-1992	INRA, Jouy-en-Josas, France	[10]	
BMG.167	ST14	3	CC3A		5	1	10	2	2	1	7	2	Fresh sheep meat (north of Tunisia)	2008	University of Tunis, Tunis, Tunisia	[43]	2065
710	ST15	3	CC3A	B	5	1	10	2	2	1	1	1	French dry-type sausage	1990-1992	INRA, Jouy-en-Josas, France	[10]	2244
AUA-MOM12.2	ST16	3	CC3A		5	1	10	1	4	3	3	4	Chilled minced beef meat	2008	Agricultural University, Athens, Greece	Unpublished	
179	ST17	3	CC3A		6	1	10	1	4	3	5	1	French dry-type sausage	1990-1992	INRA, Jouy-en-Josas, France	[10]	
184	ST18	3	CC3A		6	1	10	1	4	3	5	2	French dry-type sausage	1990-1992	INRA, Jouy-en-Josas, France	[10]	
MFPC40D14-03	ST19	3	CC3A		5	1	10	1	7	1	1	5	Vacuum Packed beef carpaccio	2009	INRA, Jouy-en-Josas, France	[31]	
BMG.168	ST20	3	CC3A		5	1	10	1	4	1	1	5	Fresh sheep meat (north of Tunisia)	2008	University of Tunis, Tunis, Tunisia	[43]	2069
MFPA43D07-02	ST20	3	CC3A		5	1	10	1	4	1	1	5	Modified-Atmosphere Packed organic beef carpaccio	2009	INRA, Jouy-en-Josas, France	[31]	
MFPC24A28-04	ST20	3	CC3A		5	1	10	1	4	1	1	5	Vacuum Packed beef carpaccio	2009	INRA, Jouy-en-Josas, France	[31]	
MFPA42D07-02	ST20	3	CC3A		5	1	10	1	4	1	1	5	Modified-Atmosphere Packed organic beef carpaccio	2009	INRA, Jouy-en-Josas, France	[31]	
MFPA44D07-02	ST20	3	CC3A		5	1	10	1	4	1	1	5	Modified-Atmosphere Packed organic beef carpaccio	2009	INRA, Jouy-en-Josas, France	[31]	
BMG.73	ST21	Admixed	singleton		5	3	10	1	4	1	2	10	Tunisian ground beef meat	2008	University of Tunis, Tunis, Tunisia	[43]	1980
BMG.51	ST21	Admixed	singleton		5	3	10	1	4	1	2	10	Fresh sheep meat (south of Tunisia)	2008	University of Tunis, Tunis, Tunisia	[43]	2006

Name of Isolate	Sequence Type	Lineage	Clade	Genotype [ref. 11]	Allelic profile								Isolation source	Date	Laboratory Collection	Reference	Genome in kb
					tuf	*recA*	*glpF*	*pepV*	*ldhL*	*rpoB*	*dnaK*	*hemN*					
MF2091	ST22	3	CC3B	B	1	1	1	2	2	3	5	14	Smoked Salmon	2004	NOFIMA, Åas, Norway	[11]	1880
BMG.126	ST23	3	CC3B		1	1	1	8	2	3	5	14	Tunisian Beef packaged sausage	2008	University of Tunis, Tunis, Tunisia	[43]	1898
MFPA22D06-02	ST23	3	CC3B		1	1	1	8	2	3	5	14	Modified-Atmosphere Packed organic beef carpaccio	2009	INRA, Jouy-en-Josas, France	[31]	
MFPB16D07-01	ST23	3	CC3B		1	1	1	8	2	3	5	14	Modified-Atmosphere Packed beef carpaccio	2009	INRA, Jouy-en-Josas, France	[31]	
MFPB17D07-03	ST23	3	CC3B		1	1	1	8	2	3	5	14	Modified-Atmosphere Packed beef carpaccio	2009	INRA, Jouy-en-Josas, France	[31]	
MF2090	ST24	3	CC3B	B	1	1	1	8	3	3	5	14	Fermented trout (Rakfisk)	2004	NOFIMA, Åas, Norway	[11]	1904
MF2089	ST24	3	CC3B	B	1	1	1	8	3	3	5	14	Fermented trout (Rakfisk)	2004	NOFIMA, Åas, Norway	[11]	1917
54	ST25	3	CC3B		1	1	1	8	2	3	20	14	French dry-type sausage	1990-1992	INRA, Jouy-en-Josas, France	[10]	
MFPA24A14-01	ST26	3	CC3B		1	1	1	2	2	9	20	14	Modified-Atmosphere Packed organic beef carpaccio	2009	INRA, Jouy-en-Josas, France	[31]	
CTC429	ST27	3	CC3B	G	1	1	1	2	5	3	16	1	Spanish dry-type sausage	1993	IRTA, Monells, Spain	[40]	2139
511	ST28	3	CC3B		1	1	10	1	2	3	21	14	Vacuum-Packed pork	1987	INRA, Jouy-en-Josas, France	[37]	2186
332	ST29	3	CC3B	I	1	1	10	8	2	6	7	12	Vacuum-Packed pork	1987	INRA, Jouy-en-Josas, France	[37]	
60	ST30	3	CC3B		1	1	10	8	2	3	19	12	French dry-type sausage	1990-1992	INRA, Jouy-en-Josas, France	[10]	
SF841	ST31	3	CC3B	D	1	1	10	4	2	3	20	12	Smoked Salmon	2006	IFREMER, Nantes, France	[32]	2057
SF843	ST31	3	CC3B	D	1	1	10	4	2	3	20	12	Smoked Salmon	2006	IFREMER, Nantes, France	[32]	2053
CRL1756	ST32	3	CC3A		3	1	10	2	2	3	18	9	Fresh anchovies	2008	CERELA, Tucumán, Argentina	Unpublished	
741	ST33	3	CC3A	B	3	1	10	2	2	9	18	8	Pork meat	1981	INRA, Jouy-en-Josas, France	[10]	2181
CRL1467	ST34	Admixed	CC3C		5	1	11	2	4	1	20	9	Argentinean-type dry sausage	2002	CERELA, Tucumán, Argentina	[11]	1954
BMG.148	ST35	Admixed	CC3C		5	1	11	2	2	1	20	9	Tunisian Fresh beef meat	2008	University of Tunis, Tunis, Tunisia	[43]	2015
SAM202	ST36	Admixed	CC3C		7	1	11	2	2	1	20	9	Greek-type dry salami	1994	National Agricultural Research Foundation, Ioannina, Greece	[44]	
SAM25	ST37	Admixed	singleton		5	1	10	2	2	3	20	17	Greek-type dry salami	1994	National Agricultural Research Foundation, Ioannina, Greece	[44]	
CTC163	ST38	Admixed	singleton	C	5	1	11	2	8	1	15	10	Spanish dry-type sausage	1993	IRTA, Monells, Spain	[40]	2301
371	ST38	Admixed	singleton		5	1	11	2	8	1	15	10	Vacuum-Packed beef	1987	INRA, Jouy-en-Josas, France	[37]	
MFPB14D06-08	ST39	Admixed	singleton		5	1	11	2	6	11	15	10	Modified-Atmosphere Packed beef carpaccio	2009	INRA, Jouy-en-Josas, France	[31]	
YMW557	ST40	Admixed	singleton	B	5	2	10	1	2	3	16	12	Fresh Meat	1986	National Institute of Health, Tokyo, Japan	[43]	2006
CTC6626	ST41	Admixed	singleton	D	5	1	10	2	7	1	13	14	Spanish dry-type sausage	1993	IRTA, Monells, Spain	[11]	1869
MFPC40D14-02	ST42	Admixed	singleton		5	1	10	2	2	9	3	9	Vacuum Packed beef carpaccio	2009	INRA, Jouy-en-Josas, France	[31]	
LTH5728	ST43	1	CC1A	I	4	4	7	8	8	8	8	18	Starter strain for German-type sausages	2000	Institut Für Libbensmitteltechnologie, Hohenheim, Germany	[11]	2147
MFPA24A14-02	ST44	1	CC1A		4	10	2	8	8	6	1	19	Modified-Atmosphere Packed organic beef carpaccio	2009	INRA, Jouy-en-Josas, France	[31]	2020
BMG.45	ST45	1	CC1A		4	10	4	8	8	6	4	19	Tunisian Beef packaged sausage	2008	University of Tunis, Tunis, Tunisia	[43]	
MFPC16A28-01	ST46	1	CC1A		4	10	4	8	8	7	4	19	Vacuum Packed beef carpaccio	2009	INRA, Jouy-en-Josas, France	[31]	
MFPA44D07-04	ST46	1	CC1A		4	10	4	8	8	7	4	19	Modified-Atmosphere Packed organic beef carpaccio	2009	INRA, Jouy-en-Josas, France	[31]	
MFPA25D06-02	ST47	1	CC1A		4	10	5	8	8	7	4	19	Modified-Atmosphere Packed organic beef carpaccio	2009	INRA, Jouy-en-Josas, France	[31]	
MFPC24D15-02	ST48	1	CC1A		4	10	8	8	8	6	9	20	Vacuum Packed beef carpaccio	2009	INRA, Jouy-en-Josas, France	[31]	
MFPB16A14-01	ST48	1	CC1A		4	10	8	8	8	6	9	20	Modified-Atmosphere Packed beef carpaccio	2009	INRA, Jouy-en-Josas, France	[31]	
MFPB16D07-02	ST49	1	CC1A		4	12	8	8	8	6	9	20	Modified-Atmosphere Packed beef carpaccio	2009	INRA, Jouy-en-Josas, France	[31]	
MFPB17D07-01	ST50	1	CC1A		4	12	8	8	8	2	9	20	Modified-Atmosphere Packed beef carpaccio	2009	INRA, Jouy-en-Josas, France	[31]	
MFPC25D15-04	ST51	1	CC1A		4	10	4	8	8	6	9	9	Vacuum Packed beef carpaccio	2009	INRA, Jouy-en-Josas, France	[31]	
MFPA25A12-02	ST52	1	CC1A		4	10	8	8	8	6	8	9	Modified-Atmosphere Packed beef carpaccio	2009	INRA, Jouy-en-Josas, France	[31]	
MFPC24A28-01	ST53	1	CC1A		4	12	8	8	8	6	6	9	Vacuum Packed beef carpaccio	2009	INRA, Jouy-en-Josas, France	[31]	
MFPC25D15-03	ST54	1	CC1A		4	10	8	8	8	6	6	21	Vacuum Packed beef carpaccio	2009	INRA, Jouy-en-Josas, France	[31]	
LTH675	ST55	1	CC1A	J	4	5	4	8	8	3	6	8	German-type sausage	1991	Institut Für Libbensmitteltechnologie, Hohenheim, Germany	[48]	2180
BMG.95	ST56	1	CC1A		4	10	4	8	8	3	8	8	Tunisian salted anchovies conserved in oil	2008	University of Tunis, Tunis, Tunisia	[43]	1951
64	ST57	1	CC1A	I	4	12	4	8	8	2	5	8	French dry-type sausage	1982	INRA, Jouy-en-Josas, France	[10]	2256
BMG.136	ST58	1	CC1A		4	10	4	8	8	6	5	8	Fresh sheep meat (west of Tunisia)	2008	University of Tunis, Tunis, Tunisia	[43]	2259
H216	ST59	1	CC1A		4	11	4	8	9	6	6	8	Fresh Meat	1986	National Institute of Health, Tokyo, Japan	[43]	
LTH677	ST60	1	CC1A	I	4	10	4	8	8	6	7	8	German-type sausage	1991	Institut Für Libbensmitteltechnologie, Hohenheim, Germany	[48]	2309
CTC6429	ST61	1	CC1A	I	4	10	2	8	8	6	7	8	Spanish dry-type sausage	1993	IRTA, Monells, Spain	[11]	2278
33	ST62	1	CC1A	I	4	7	4	8	8	7	7	8	French dry-type sausage	1990-1992	INRA, Jouy-en-Josas, France	[10]	2103
BMG.120	ST63	1	CC1A		4	10	9	8	8	6	6	8	Tunisian Beef merguez	2008	University of Tunis, Tunis, Tunisia	[43]	1951
R29836	ST64	1	CC1A		4	10	9	8	8	6	5	9	Traditionnal Belgian sourdough	2008	Vrij Universiteit, Brussels, Belgium	[45]	
R29849	ST64	1	CC1A		4	10	9	8	8	6	5	9	Traditionnal Belgian sourdough	2008	Vrij Universiteit, Brussels, Belgium	[45]	
AUA-20087	ST64	1	CC1A		4	10	9	8	8	6	5	9	Industrial dry-type Greek sausages	2004	Agricultural University, Athens, Greece	Unpublished	

Name of Isolate	Sequence Type	Lineage	Clade	Genotype [ref. 11]	Allelic profile								Isolation source	Date	Laboratory Collection	Reference	Genome in kb
					tuf	*recA*	*glpF*	*pepV*	*ldhL*	*rpoB*	*dnaK*	*hemN*					
CRL1755	ST65	1	CC1A		4	10	9	8	8	6	6	9	Fresh anchovies	2008	CERELA, Tucumán, Argentina	Unpublished	
MFPB16D07-04	ST66	1	CC1A		4	10	4	8	8	1	6	9	Modified-Atmosphere Packed beef carpaccio	2009	INRA, Jouy-en-Josas, France	[31]	
BMG.107	ST67	Admixed	singleton		4	10	9	8	4	3	6	9	Tunisian Turkey packaged sausage with garlic	2008	University of Tunis, Tunis, Tunisia	[43]	2090
MFPA29A14-01	ST68	1	singleton		4	5	4	7	7	5	5	2	Modified-Atmosphere Packed organic beef carpaccio	2009	INRA, Jouy-en-Josas, France	[31]	
MFPC40A28-04	ST69	Admixed	singleton		5	5	1	2	7	3	5	2	Vacuum Packed beef carpaccio	2009	INRA, Jouy-en-Josas, France	[31]	
LTH2070	ST70	Admixed	singleton	I	4	5	4	5	4	1	11	2	Sauerkraut	Not known	Institut Für Libbensmitteltechnologie, Hohenheim, Germany	[11]	2150
LTH2076	ST71	Admixed	singleton		4	6	4	5	4	1	11	2	Sauerkraut	Not known	Institut Für Libbensmitteltechnologie, Hohenheim, Germany	[11]	
ATCC15521	ST72	1	CC1A	J	4	10	4	8	8	6	6	2	Fermented sake beverage	1934	American Type Culture Collection		1925
LTH938	ST72	1	CC1A		4	10	4	8	8	6	6	2	German-type sausage	1991	Institut Für Libbensmitteltechnologie, Hohenheim, Germany	[48]	
175	ST72	1	CC1A		4	10	4	8	8	6	6	2	French dry-type sausage	1990-1992	INRA, Jouy-en-Josas, France	[10]	
R29873	ST73	1	CC1A		4	10	9	8	8	6	6	2	Traditionnal Belgian sourdough	2008	Vrij Universiteit, Brussels, Belgium	[45]	
MFPA43D07-01	ST73	1	CC1A		4	10	9	8	8	6	6	2	Modified-Atmosphere Packed organic beef carpaccio	2009	INRA, Jouy-en-Josas, France	[31]	
MFPA25D06-01	ST73	1	CC1A		4	10	9	8	8	6	6	2	Modified-Atmosphere Packed organic beef carpaccio	2009	INRA, Jouy-en-Josas, France	[31]	
MFPA19A15-01	ST74	1	CC1A		4	8	5	8	8	6	6	2	Modified-Atmosphere Packed organic beef carpaccio	2009	INRA, Jouy-en-Josas, France	[31]	
TISTR890	ST75	1	CC1A	J	4	7	4	8	8	6	6	2	Taiwanese pork Nam product	1983	Mahidol University, Bangkok, Thaïland	[10]	1878
BMG.101	ST76	1	CC1A		4	10	4	8	8	1	6	7	Tunisian Turkey packaged ham	2008	University of Tunis, Tunis, Tunisia	[43]	
31	ST77	Admixed	singleton		5	10	10	6	4	1	1	6	French dry-type sausage	1990-1992	INRA, Jouy-en-Josas, France	[10]	
BMG.37	ST78	Admixed	singleton		5	13	10	2	7	9	5	2	Fresh sheep meat (east of Tunisia)	2008	University of Tunis, Tunis, Tunisia	[43]	1897
MFPA17D07-02	ST79	2	CC2D		5	12	10	2	4	1	10	14	Modified-Atmosphere Packed organic beef carpaccio	2009	INRA, Jouy-en-Josas, France	[31]	
CTC335	ST80	2	CC2D	E	5	12	11	2	4	1	14	10	Spanish dry-type sausage	1993	IRTA, Monells, Spain	[40]	1952
SF770	ST81	2	CC2D	E	5	12	11	2	4	1	10	12	Smoked Salmon	2006	IFREMER, Nantes, France	[32]	2077
SF842	ST81	2	CC2D	E	5	12	11	2	4	1	10	12	Smoked Salmon	2006	IFREMER, Nantes, France	[32]	2020
SF771	ST81	2	CC2D	E	5	12	11	2	4	1	10	12	Smoked Salmon	2006	IFREMER, Nantes, France	[32]	
MFPA29D07-02	ST82	2	CC2C		5	12	10	2	2	1	3	9	Modified-Atmosphere Packed organic beef carpaccio	2009	INRA, Jouy-en-Josas, France	[31]	
MFPC40D14-04	ST83	2	CC2C		5	13	10	2	4	9	3	9	Vacuum Packed beef carpaccio	2009	INRA, Jouy-en-Josas, France	[31]	
MFPC40A28-03	ST83	2	CC2C		5	13	10	2	4	9	3	9	Vacuum Packed beef carpaccio	2009	INRA, Jouy-en-Josas, France	[31]	
MFPA42A14-05	ST83	2	CC2C		5	13	10	2	4	9	3	9	Modified-Atmosphere Packed organic beef carpaccio	2009	INRA, Jouy-en-Josas, France	[31]	
FLEC02	ST84	2	CC2C		5	13	10	2	2	9	3	9	Modified-Atmosphere Packed beef carpaccio	2008	INRA, Jouy-en-Josas, France	Unpublished	
MFPB42D06-02	ST84	2	CC2C		5	13	10	2	2	9	3	9	Modified-Atmosphere Packed beef carpaccio	2008	INRA, Jouy-en-Josas, France	[31]	
381	ST85	2	CC2C		5	13	10	2	4	2	3	9	Vacuum-Packed pork	1987	INRA, Jouy-en-Josas, France	[37]	
160*1	ST86	2	CC2C	C	5	13	10	2	2	2	3	9	Horse meat	1982	INRA, Jouy-en-Josas, France	[10]	1890
LTH5590	ST86	2	CC2C	C	5	13	10	2	2	2	3	9	Human feces	2002	Institut Für Libbensmitteltechnologie, Hohenheim, Germany	[39]	1915
BMG.106	ST86	2	CC2C		5	13	10	2	2	2	3	9	Tunisian Turkey packaged sausage with garlic	2008	University of Tunis, Tunis, Tunisia	[43]	1895
AGR48	ST86	2	CC2C	C	5	13	10	2	2	2	3	9	Chilled lamb meat	2007	AgResearch, Hamilton, New-Zealand	[11]	1943
CTC014	ST86	2	CC2C	C	5	13	10	2	2	2	3	9	Spanish dry-type sausage	1993	IRTA, Monells, Spain	[40]	2038
BMG.164	ST86	2	CC2C		5	13	10	2	2	2	3	9	Tunisian dried salted Octopus	2008	University of Tunis, Tunis, Tunisia	[47]	1866
GV1	ST86	2	CC2C		5	13	10	2	2	2	3	9	Dry cured ham	1981	INRA, Jouy-en-Josas, France	[10]	
HC4	ST86	2	CC2C		5	13	10	2	2	2	3	9	Ground-beef	1991	INRA, Jouy-en-Josas, France	[10]	
BMG.170	ST86	2	CC2C		5	13	10	2	2	2	3	9	Tunisian Fresh pork meat	2008	University of Tunis, Tunis, Tunisia	[43]	1886
MFPB29D07-01	ST86	2	CC2C		5	13	10	2	2	2	3	9	Modified-Atmosphere Packed beef carpaccio	2008	INRA, Jouy-en-Josas, France	[31]	
378	ST87	2	CC2D		5	12	11	8	4	1	3	10	Vacuum-Packed pork	1987	INRA, Jouy-en-Josas, France	[37]	
MFPA25A12-06	ST88	2	singleton		8	10	10	6	4	3	20	15	Modified-Atmosphere Packed organic beef carpaccio	2009	INRA, Jouy-en-Josas, France	[31]	
AGR47	ST89	2	singleton		5	10	10	6	4	1	21	14	Chilled lamb meat	2007	AgResearch, Hamilton, New-Zealand	[11]	
AGR51	ST89	2	singleton	H	5	10	10	6	4	1	21	14	Chilled lamb meat	2007	AgResearch, Hamilton, New-Zealand	[11]	2038
14	ST90	Admixed	singleton	G	4	5	10	8	2	1	21	14	French dry-type sausage	1990-1992	INRA, Jouy-en-Josas, France	[10]	2044
18	ST90	Admixed	singleton	G	4	5	10	8	2	1	21	14	French dry-type sausage	1990-1992	INRA, Jouy-en-Josas, France	[10]	2053
AUA-M5M8.6	ST91	2	singleton		5	13	10	2	4	9	20	12	Chilled minced beef meat	2008	Agricultural University, Athens, Greece	Unpublished	
21	ST92	2	singleton	J	5	10	10	8	4	9	20	14	French dry-type sausage	1990-1992	INRA, Jouy-en-Josas, France	[10]	2043
MFPA42D07-04	ST93	2	singleton		1	9	10	2	7	10	16	16	Modified-Atmosphere Packed organic beef carpaccio	2009	INRA, Jouy-en-Josas, France	[31]	
495	ST94	2	singleton	E	1	12	10	2	8	10	16	16	Vacuum-Packed pork	1987	INRA, Jouy-en-Josas, France	[37]	1887
YMW540	ST95	Admixed	singleton	E	1	12	5	2	7	10	16	14	Fresh Meat	1986	National Institute of Health, Tokyo, Japan	[43]	2040
MF1053	ST96	Admixed	singleton		1	12	10	2	2	6	17	2	Fermented trout (Rakfisk)	2004	NOFIMA, Åas, Norway	[50]	2045
Lb674	ST96	Admixed	singleton		1	12	10	2	2	6	17	2	Fresh meat	1989	Federal Center For Meat Research, Kulmbach, Germany	[50]	2038

Name of Isolate	Sequence Type	Lineage	Clade	Genotype [ref. 11]	Allelic profile								Isolation source	Date	Laboratory Collection	Reference	Genome in kb
					tuf	*recA*	*glpF*	*pepV*	*ldhL*	*rpoB*	*dnaK*	*hemN*					
MFPB42D06-01	ST97	Admixed	singleton		1	12	10	3	7	12	16	3	Modified-Atmosphere Packed beef carpaccio	2008	INRA, Jouy-en-Josas, France	[31]	
BMG.40	ST98	Admixed	singleton		2	5	10	2	4	3	20	2	Tunisian Beef packaged salami	2008	University of Tunis, Tunis, Tunisia	[43]	1900
G3	ST99	2	CC2B	D	6	12	10	2	4	1	20	12	Ground beef	1990	INRA, Jouy-en-Josas, France	[10]	2094
AGR53	ST99	2	CC2B	F	6	12	10	2	4	1	20	12	Chilled lamb meat	2007	AgResearch, Hamilton, New-Zealand	[11]	2116
JOUY-181	ST99	2	CC2B		6	12	10	2	4	1	20	12	French dry-type sausage	1990-1992	INRA, Jouy-en-Josas, France	[10]	
LTH1764	ST99	2	CC2B	D	6	12	10	2	4	1	20	12	Sauerkraut	Not known	Institut Für Libbensmitteltechnologie, Hohenheim, Germany	[11]	2143
S9	ST99	2	CC2B		6	12	10	2	4	1	20	12	Ground beef	1985	INRA, Jouy-en-Josas, France	[10]	
MFPB19A15-01	ST99	2	CC2B		6	12	10	2	4	1	20	12	Modified-Atmosphere Packed beef carpaccio	2008	INRA, Jouy-en-Josas, France	[31]	
Lb706	ST100	2	CC2B	F	6	12	10	2	4	3	20	12	Fresh meat	1989	Federal Center For Meat Research, Kulmbach, Germany	[50]	2081
Lb790x	ST101	2	CC2B		6	12	2	2	4	3	20	12	Fresh meat	1989	Federal Center For Meat Research, Kulmbach, Germany	[50]	1907
MFPA42A14-01	ST102	2	CC2B		6	12	2	2	4	3	20	13	Modified-Atmosphere Packed organic beef carpaccio	2009	INRA, Jouy-en-Josas, France	[31]	
LTH939	ST103	2	CC2B		6	12	3	2	4	3	20	12	German-type sausage	1991	Institut Für Libbensmitteltechnologie, Hohenheim, Germany	[48]	
156	ST104	2	CC2B	H	6	12	10	2	4	4	20	12	French dry-type sausage	1990-1992	INRA, Jouy-en-Josas, France	[10]	2205
LV82	ST104	2	CC2B		6	12	10	2	4	4	20	12	Bacon	1983	Meat Research Institute, Bristol, UK	[47]	
BMG.105	ST104	2	CC2B		6	12	10	2	4	4	20	12	Tunisian Beef packaged sausage	2008	University of Tunis, Tunis, Tunisia	[43]	2094
AGR46	ST104	2	CC2B	H	6	12	10	2	4	4	20	12	Chilled lamb meat	2007	AgResearch, Hamilton, New-Zealand	[11]	1884
SAM129	ST104	2	CC2B		6	12	10	2	4	4	20	12	Greek-type dry salami	1994	National Agricultural Research Foundation, Ioannina, Greece	[48]	
174	ST104	2	CC2B		6	12	10	2	4	4	20	12	French dry-type sausage	1990-1992	INRA, Jouy-en-Josas, France	[10]	
LTH945	ST104	2	CC2B		6	12	10	2	4	4	20	12	German-type sausage	1991	Institut Für Libbensmitteltechnologie, Hohenheim, Germany	[48]	
BMG.138	ST104	2	CC2B		6	12	10	2	4	4	20	12	Fresh sheep meat (west of Tunisia)	2008	University of Tunis, Tunis, Tunisia	[43]	2098
BMG.115	ST104	2	CC2B		6	12	10	2	4	4	20	12	Tunisian Sheep dried meat	2008	University of Tunis, Tunis, Tunisia	[43]	1973
LV21	ST104	2	CC2B	F	6	12	10	2	4	4	20	12	Vacuum-packed pork meat	1983	Meat Research Institute, Bristol, UK	[47]	1847
CTC494	ST104	2	CC2B	B	6	12	10	2	4	4	20	12	Spanish dry-type sausage	1993	IRTA, Monells, Spain	[40]	1851
SAM148	ST104	2	CC2B		6	12	10	2	4	4	20	12	Greek-type dry salami	1994	National Agricultural Research Foundation, Ioannina, Greece	[44]	
AGR42	ST104	2	CC2B		6	12	10	2	4	4	20	12	Chilled lamb meat	2007	AgResearch, Hamilton, New-Zealand	[11]	
ADIV-IM8	ST104	2	CC2B		6	12	10	2	4	4	20	12	Traditional dry-type French Sausage	Not known	ADIV, Clermont-Ferrand, France	Unpublished	
ADIV-L2	ST104	2	CC2B		6	12	10	2	4	4	20	12	Industrial dry-type French Sausage	Not known	ADIV, Clermont-Ferrand, France	Unpublished	
MFPA29A14-04	ST104	2	CC2B		6	12	10	2	4	4	20	12	Modified-Atmosphere Packed organic beef carpaccio	2009	INRA, Jouy-en-Josas, France	[31]	
MFPA29D07-03	ST104	2	CC2B		6	12	10	2	4	4	20	12	Modified-Atmosphere Packed organic beef carpaccio	2009	INRA, Jouy-en-Josas, France	[31]	
MF2088	ST105	2	CC2B	B	6	12	12	2	4	4	20	12	Fermented trout (Rakfisk)	2004	NOFIMA, Åas, Norway	[11]	1884
MFPB17D07-07	ST106	2	2B		9	12	10	2	4	4	20	12	Modified-Atmosphere Packed beef carpaccio	2009	INRA, Jouy-en-Josas, France	[30]	
CTC041	ST107	2	CC2A	A	5	12	5	2	6	1	15	10	Spanish dry-type sausage	1993	IRTA, Monells, Spain	[40]	2010
TISTR911	ST107	2	CC2A	A	5	12	5	2	6	1	15	10	Taiwanese pork Nam product	1983	Mahidol University, Bangkok, Thaïland	[10]	1856
504	ST107	2	CC2A	A	5	12	5	2	6	1	15	10	Vacuum-Packed pork	1987	INRA, Jouy-en-Josas, France	[37]	1838
YME344	ST107	2	CC2A	A	5	12	5	2	6	1	15	10	Fresh Meat	1986	National Institute of Health, Tokyo, Japan	[43]	1964
CTC287	ST107	2	CC2A	A	5	12	5	2	6	1	15	10	Spanish dry-type sausage	1993	IRTA, Monells, Spain	[40]	1884
AUA-605	ST107	2	CC2A		5	12	5	2	6	1	15	10	Industrial dry-type Greek sausages	2007	Agricultural University, Athens, Greece	Unpublished	1814
AUA-737	ST107	2	CC2A		5	12	5	2	6	1	15	10	Industrial dry-type Greek sausages	2007	Agricultural University, Athens, Greece	Unpublished	
CTC427	ST107	2	CC2A	A	5	12	5	2	6	1	15	10	Spanish dry-type sausage	1993	IRTA, Monells, Spain	[40]	
MFPC16D14-01	ST107	2	CC2A		5	12	5	2	6	1	15	10	Vacuum Packed beef carpaccio	2009	INRA, Jouy-en-Josas, France	[31]	
ADIV-L152	ST108	2	CC2A		5	12	5	2	7	1	15	10	Farm dry-type French Sausage	Not known	ADIV, Clermont-Ferrand, France	Unpublished	
MFPC14D14-01	ST108	2	CC2A		5	12	5	2	7	1	15	10	Vacuum Packed beef carpaccio	2009	INRA, Jouy-en-Josas, France	[31]	
MFPC15D14-02	ST108	2	CC2A		5	12	5	2	7	1	15	10	Vacuum Packed beef carpaccio	2009	INRA, Jouy-en-Josas, France	[31]	
MFPA22D06-01	ST108	2	CC2A		5	12	5	2	7	1	15	10	Modified-Atmosphere Packed organic beef carpaccio	2009	INRA, Jouy-en-Josas, France	[31]	
MFPB15D06-04	ST108	2	CC2A		5	12	5	2	7	1	15	10	Modified-Atmosphere Packed beef carpaccio	2009	INRA, Jouy-en-Josas, France	[31]	
MFPC14A29-01	ST108	2	CC2A		5	12	5	2	7	1	15	10	Vacuum Packed beef carpaccio	2009	INRA, Jouy-en-Josas, France	[31]	
MFPA15A12-01	ST108	2	CC2A		5	12	5	2	7	1	15	10	Modified-Atmosphere Packed organic beef carpaccio	2009	INRA, Jouy-en-Josas, France	[31]	
MFPC24D15-03	ST108	2	CC2A		5	12	5	2	7	1	15	10	Vacuum Packed beef carpaccio	2009	INRA, Jouy-en-Josas, France	[31]	
MFPB14D06-01	ST108	2	CC2A		5	12	5	2	7	1	15	10	Modified-Atmosphere Packed beef carpaccio	2009	INRA, Jouy-en-Josas, France	[31]	
300	ST109	2	CC2A	A	5	12	5	2	4	11	15	10	Vacuum-Packed beef	1987	INRA, Jouy-en-Josas, France	[37]	1934
518	ST109	2	CC2A		5	12	5	2	4	11	15	10	Vacuum-Packed pork	1987	INRA, Jouy-en-Josas, France	[37]	
SAM526	ST110	2	CC2A		5	12	6	2	2	11	15	10	Greek-type dry salami	1994	National Agricultural Research Foundation, Ioannina, Greece	[44]	
MFPC41A28-07	ST111	2	CC2A		5	12	5	2	2	11	15	10	Vacuum Packed beef carpaccio	2009	INRA, Jouy-en-Josas, France	[31]	

Name of Isolate	Sequence Type	Lineage	Clade	Genotype [ref. 11]	Allelic profile								Isolation source	Date	Laboratory Collection	Reference	Genome in kb
					tuf	*recA*	*glpF*	*pepV*	*ldhL*	*rpoB*	*dnaK*	*hemN*					
MFPC14A29-04	ST111	2	CC2A		5	12	5	2	2	11	15	10	Vacuum Packed beef carpaccio	2009	INRA, Jouy-en-Josas, France	[31]	
AUA-MOM4.1	ST112	2	CC2A		5	12	5	2	6	11	12	10	Chilled minced beef meat	2008	Agricultural University, Athens, Greece	Unpublished	
ADIV-LB10	ST113	2	CC2A		5	12	5	2	6	11	15	11	Fresh pork meat from industrial producer	2005	ADIV, Clermont-Ferrand, France	Unpublished	
LV5	ST114	2	CC2A	A	5	12	5	2	6	11	15	10	Vacuum-packed pork meat	1983	Meat Research Institute, Bristol, UK	[47]	1911
557	ST114	2	CC2A		5	12	5	2	6	11	15	10	Vacuum-Packed pork	1987	INRA, Jouy-en-Josas, France	[37]	
CRL1758	ST114	2	CC2A		5	12	5	2	6	11	15	10	Fresh anchovies	2008	CERELA, Tucumán, Argentina	Unpublished	
MFPA24D07-01	ST114	2	CC2A		5	12	5	2	6	11	15	10	Modified-Atmosphere Packed organic beef carpaccio	2009	INRA, Jouy-en-Josas, France	[31]	
MFPB29A14-05	ST114	2	CC2A		5	12	5	2	6	11	15	10	Modified-Atmosphere Packed beef carpaccio	2009	INRA, Jouy-en-Josas, France	[31]	
MFPC24D15-01	ST114	2	CC2A		5	12	5	2	6	11	15	10	Vacuum Packed beef carpaccio	2009	INRA, Jouy-en-Josas, France	[31]	
MFPC24A28-02	ST114	2	CC2A		5	12	5	2	6	11	15	10	Vacuum Packed beef carpaccio	2009	INRA, Jouy-en-Josas, France	[31]	
MFPC23A29-01	ST114	2	CC2A		5	12	5	2	6	11	15	10	Vacuum Packed beef carpaccio	2009	INRA, Jouy-en-Josas, France	[31]	
MFPA25A12-01	ST114	2	CC2A		5	12	5	2	6	11	15	10	Modified-Atmosphere Packed organic beef carpaccio	2009	INRA, Jouy-en-Josas, France	[31]	
72	ST115	2	CC2A	A	5	12	5	2	6	12	3	10	French dry-type sausage	1990-1992	INRA, Jouy-en-Josas, France	[10]	1826
23K	ST116	2	CC2A		5	12	5	2	6	12	15	10	French dry-type sausage	1990-1992	INRA, Jouy-en-Josas, France	[10]	1886
LV34	ST116	2	CC2A	A	5	12	5	2	6	12	15	10	Vacuum-packed beef meat	1983	Meat Research Institute, Bristol, UK	[47]	1903
V553	ST116	2	CC2A	A	5	12	5	2	6	12	15	10	Fresh Meat	1986	National Institute of Health, Tokyo, Japan	[43]	1905
MFPA25A12-08	ST116	2	CC2A		5	12	5	2	6	12	15	10	Modified-Atmosphere Packed organic beef carpaccio	2009	INRA, Jouy-en-Josas, France	[31]	
MFPA17D07-01	ST116	2	CC2A		5	12	5	2	6	12	15	10	Modified-Atmosphere Packed organic beef carpaccio	2009	INRA, Jouy-en-Josas, France	[31]	
MFPC14D14-04	ST116	2	CC2A		5	12	5	2	6	12	15	10	Vacuum Packed beef carpaccio	2009	INRA, Jouy-en-Josas, France	[31]	
MFPB11D07-03	ST116	2	CC2A		5	12	5	2	6	12	15	10	Modified-Atmosphere Packed beef carpaccio	2009	INRA, Jouy-en-Josas, France	[31]	
