# Supplementary material for: Population Genetics of Lactobacillus sakei Reveals Three Lineages with Distinct Evolutionary Histories
Source: PLoS One. 2013 Sep 19;8(9):e73253. doi: 10.1371/journal.pone.0073253 (PMC3777942; doi:10.1371/journal.pone.0073253)
Supplement: Table S2 — Description of primers used in the MLST analysis. (DOC) [file pone.0073253.s005.doc]

Table S2 : Primers used for the MLST analysis.

| Gene | Primer's name | Sequence 5'-3' | GC % |
| --- | --- | --- | --- |
| *ldhL* | LDHL-MLST14-F  LDHL-MLST14-R | TTGGCAAACATTGAAAAAGATCACC  ACCGATACCGTAGAATGTAGCACCT | 36  48 |
| *pepV* | PEPV-MLST4-F  PEPV-MLST4-R | ATAAGGATCAAATGCTTACCGAC  CATTGCACCAGTTAAGATTGCC | 39  45 |
| *glpF* | GLPF-MLST6-F  GLPF-MLST6-R | TAATGGTCCAAGCACTAGGCG  GTGTCAATGCTAACAATGCGAA | 52  41 |
| *Tuf* | TUF-MLST9-F  TUF-MLST9-R | CAGAATACGATTTCCCTGGTGA  TTTGTGAGTTTGGATTGAACCA | 45  36 |
| *rpoB* | RPOB-MLST16-F  RPOB-MLST16-R | ACGATTTAGCTTCTGTTGGTCG  GTTCCATCCGTGATAAACCGATCC | 45  50 |
| *dnaK* | DNAK-MLST10-F  DNAK-MLST10-R | GATTATCTTGGTGAAGAAGTCACA  TTGTACGATCAACTAAGTCTGATG | 38  38 |
| *recA* | RECA-MLST5-F  RECA-MLST5-R | CAAGTGGGGCCGTTGATATT  GTTCTGAGCCATAAGAGTACCA | 50  45 |
| *hemN* | HEMN-MLST11-F  HEMN-MLST11-R | CGCTTGAGCATTGGCGTGCAG  CAAAGCCGAAATACTTTTCATTCC | 62  38 |
